# Figuring out what is happening: the discovery of two electrophysiological phenomena

**DOI:** 10.1007/s40656-022-00502-1

**Published:** 2022-05-17

**Authors:** William Bechtel, Richard Vagnino

**Affiliations:** Department of Philosophy, University of California, San Diego, La Jolla, CA 92093-0119 USA

**Keywords:** Scientific phenomena, Mechanistic explanation, Membrane potential, Action potential, History of electrophysiology

## Abstract

Research devoted to characterizing phenomena is underappreciated in philosophical accounts of scientific inquiry. This paper develops a diachronic analysis of research over 100 years that led to the recognition of two related electrophysiological phenomena, the membrane potential and the action potential. A diachronic perspective allows for reconciliation of two threads in philosophical discussions of phenomena—Hacking’s treatment of phenomena as manifest in laboratory settings and Bogen and Woodward’s construal of phenomena as regularities in the world. The diachronic analysis also reveals the epistemic tasks that contribute to establishing phenomena, including the development of appropriate investigative techniques and concepts for characterizing them.

## Introduction

Philosophers of biology have focused extensively on explanation, especially on characterizing mechanistic explanations (Bechtel & Abrahamsen, 2005; Glennan, 2017; Machamer et al., 2000) and on analyzing how they are discovered (Bechtel & Richardson, 1993/2010; Craver & Darden, 2013). Following Bogen and Woodward (1988), explanations are viewed as explaining phenomena. However, scientific research that is directed simply at determining what the phenomena are—on figuring out what is happening— has received little attention until recently.^1^ Feest (2017) has pioneered efforts at characterizing how phenomena are discovered, emphasizing a process she calls *stabilization*. Her account of phenomena draws upon both Bogen and Woodward (1988) and Hacking (1983) and reveals a tension between their accounts. Hacking argues that phenomena seldom, if ever, simply occur in nature but rather in artificial, highly idiosyncratic laboratory setups and only when researchers bring a substantial measure of skill and patience to their production. Bogen and Woodward, in contrast, treat phenomena as part “of the natural order itself” (p. 321). Feest attempts to reconcile this difference by invoking two related and more fine-grained concepts (*surface* and *hidden* phenomena) (p. 58). By focusing on a temporarily extended period of research in which two related phenomena—the membrane potentials and the action potentials of nerves and muscles—were established, we propose a diachronic resolution to the tension. Early in the investigation of a potential phenomenon, it remains closely associated with experimental tools and techniques that are first used to identify and manipulate it. As time passes, and the repertoire of experimental protocols in which it can be made manifest expands, scientists develop concepts that increasingly abstract the fledging phenomenon from any one experimental context, resulting in it having the appearance of something “external.” This trajectory involves both developing new means to investigate the phenomenon and new conceptual tools for characterizing it.

We have selected for our focus research that led to the acceptance of two related phenomena: what are now recognized as the membrane potential (the maintenance in all cells, but especially muscle and nerve cells, of an electrical potential across the membrane) and the action potential (a reversal followed by restoration of the membrane potential that propagates along muscles and neurons).^2^ Even though his characterizations would undergo significant revisions throughout the twentieth century, one can recognize the modern phenomena of the membrane potential and the action potential in the research of Julius Bernstein at the beginning of the century. These phenomena had no place, however, in the investigations in the seventeenth and most of the eighteenth century as researchers began to explore the effects of electricity on living organisms and the electric shocks produced by fish such as eels and rays (which had first been reported by the ancient Greeks and Egyptians). The membrane potential intruded in the work of Luigi Galvani in the 1780s and the action potential in the studies of Carlo Matteucci in the 1830s. Both, however, characterized what they encountered using concepts very different than those employed today. Between then and the research of Bernstein, numerous investigators introduced new research instruments and experimental protocols, generated new findings, and offered different conceptualizations of the phenomena. By examining these, we offer an account of the epistemic work involved in establishing scientific phenomena as accepted happenings in the natural world.

Following on the initial discussions by Hacking and by Bogen and Woodward, several philosophers have drawn attention to the epistemic efforts needed to establish phenomena. Sullivan (2008, 2010), in particular, has drawn attention to the importance of specific experimental protocols and how the differences between them can result in scientists actually studying different phenomena. Recognizing this, Feest (2010) emphasizes the challenge of regularizing new phenomena in the development of experimental protocols and especially in developing new concepts with which to characterize the phenomena. This involves both adapting concepts from other nearby domains by way of analogy and operationalizing them in terms of the researcher’s experimental protocols. Once the new concepts and experimental protocols are developed, the phenomena may be accepted as regular features of the world.

An advantage of focusing on examples in which these activities played out over a prolonged period is that it is easier to identify the epistemic work required to establish new phenomena. We are, of course, not the first to arrive at this insight. Rheinberger’s (1997) seminal work on protein synthesis and the “emergence” of transfer RNA in the laboratories at Huntington Memorial Hospital serves as a touchstone in this regard, both for its extended historical outlook, and its focus on the creation and development of “epistemic things” which are the “material entities or processes — physical structures, chemical reactions, biological functions — that constitute the objects of inquiry” (p. 28). Epistemic things, Rheinberger suggests, often begin their careers as unexpected intrusions or “recalcitrant ‘noise’” before making the leap to becoming objects of scientific interest (p. 21). In time, they may lose their status as objects of interest in their own right and transition to “technical objects,” which in turn support further investigation into other targets. The slide from epistemic thing to technical object (and sometimes back again) is a trope that shows up throughout the history we recount. It is, in fact, the dynamic nature of these entities—and the particular ways in which they leave their traces in the experimental setups in which they are embedded—that we argue is key to resolving the tension between the differing conceptions of phenomena identified by Feest. While our analysis of the history of the membrane potential and the action potential parallels Rheinberger’s analysis in adopting a diachronic perspective and draws entensively on his analysis, an important difference is that the scientists he describes were engaged in the quest for explanation of an already identified phenomenon—protein synthesis. While the desire to explain is often manifest in the scientists we discuss, we focus our analysis on their quest to characterize phenomena themselves. Only by the end of the more than 100-year endeavor we describe were researchers in a position to say that they were explaining the membrane potential or the action potential.

In the final section, we return to the question of how adopting a diachronic perspective allows us to understand in greater resolution how scientists in this case, and potentially in others, came to identify new phenomena. In the two intervening sections we analyze the historical process in which the membrane potential and the action potential came to be recognized as scientific phenomena. The two phenomena are clearly related—the action potential involves the reversal and restoration of the membrane potential. The same set of researchers played critical roles in developing the understanding of each. Nonetheless, research leading to the characterization of the membrane potential can be disentangled from that devoted to the action potential and so we present it first in Sect. 2, analyzing the main developments between Galvani’s characterization of what he termed *animal electricity* and Bernstein’s account of what he termed the *membrane potential*. Then, in Sect. 3 we focus on the developments between Matteucci’s observation of the termination of the muscle current in tetanus and Bernstein’s account of the action potential as a deflection in the membrane potential that could be propagated in nerves and muscle.

## From animal electricity to the membrane potential

Despite the significance of his work, Galvani was far from the first to investigate electrical phenomena in animals. The seventeenth and eighteenth centuries witnessed a growing interest in electrical phenomena, promoted in part by the development of machines that would generate electrical changes (for a detailed recent historical analysis of the investigations summarized in this and the next paragraph, see Schiffer, 2003; for earlier treatments, see Hoff, 1936; Walker, 1937). Otto von Guericke developed the first electrostatic generator, consisting of a sulfur globe attached to an iron rod. Other electrical devices soon followed, including the Leyden jar, a forerunner to the modern capacitor that stores electrical charges. The glass of the jar acted as an insulator, which was coated on the outside with lead or tin foil. Inside, a chain made of brass (or another metal, such as lead shot) was connected to a wire that extended out through a stopper. When the wire was connected to one conductor of an electrostatic generator and the other was grounded, operation of the machine would cause a charge, often amounting to thousands of volts, to collect and endure for periods of hours or days until released as a shock when the circuit was completed.

Schiffer (2003, chapter 6) relates how a number of investigators began to explore the physiological effects of electric shocks both on plants (e.g., accelerating the germination of seeds or flowering of plants) and animals (increased blood flow or weight loss). Physicians explored the use of electrical shocks as therapies for conditions such as paralysis. Other researchers focused on animals as a source of electricity—including fish that had been known from antiquity to produce shocks (Turkel, 2013). John Walsh explicitly identified the shocks as electrical, and was among the first to investigate how they were produced. One consequence of this research was to convince some who had been initially skeptical to accept that nerves transmitted electricity (Piccolino & Bresadola, 2013); however, plenty of skeptics remained (Kipnis, 2005).

### Galvani’s finding of animal electricity

Among the noted effects of applying electric shocks to animals was muscle contraction. This provided the starting point for Galvani’s research that led to his conclusion that muscles themselves stored electricity much as a Leyden jar did. Galvani conducted most of his studies on frogs, already a widely used experimental animal by the 1780s when he began his experiments.^3^ Frogs offered several advantages: their nerves were easily identified and separated, and their muscles produced easily detectable contractions for a prolonged period after the animal had been killed. Galvani developed a preparation in which the hind limbs were cut off below the forelimbs, skinned, and disemboweled, with the crural nerve extending out, sometimes in conjunction with the spinal cord. As he standardized his procedure for preparing frogs, he came to refer to them as *frogs prepared in the usual manner.* One of the first observations Galvani reported was that “very little electrical fluid—which is very far to produce the least electric sign—is sufficient to produce the contractions” (Galvani, 1937, Piccolini and Bresadola (2013), p. 79).

**Fig. 1:**
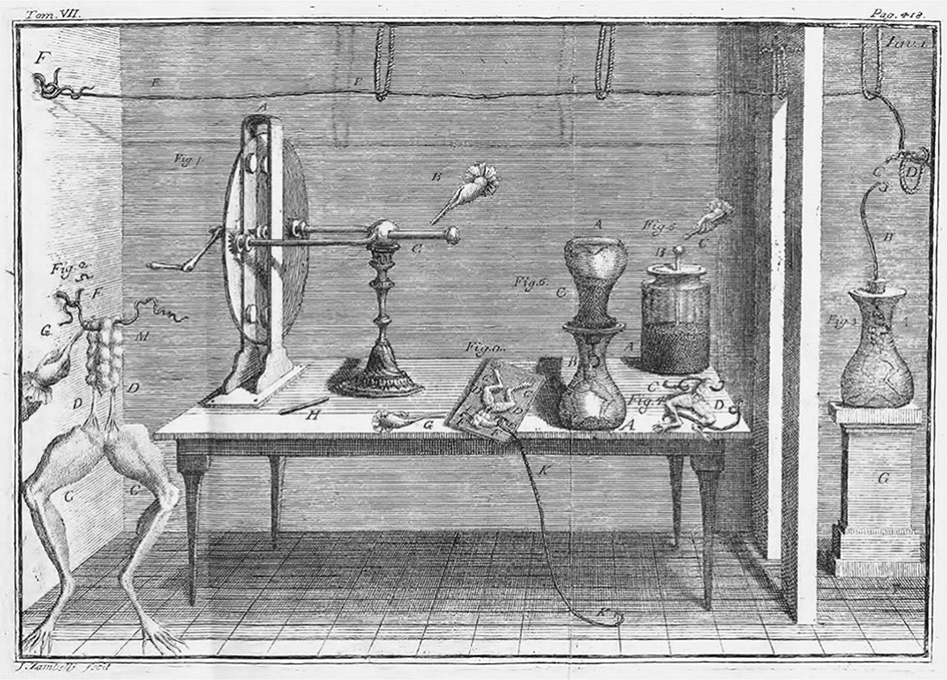
Plate 1 from *De Viribus* showing the spark generator on the left of the table and a Leyden jar in the back right corner. On the far left is inserted an image of an experimenter touching the frog leg with a scalpel. The other components show various preparation of frog legs. On the far right the leg is contained in a glass vesicle, used to insulate it from any electrical charges in the vicinity

In *De viribus electricitatis* Galvani (1791; passages quoted in what follows are from the translation by Green, 1953) described selected experiments that he conducted between 1780 and 1790.^4^ Even though the account is abridged, it testifies to how he explored a wide range of conditions so as to determine which would give rise to muscle contractions. *De viribus* begins with the revelation that directly shocking the frog was unnecessary to produce contractions: when one assistant generated a spark from an electrostatic machine while another, at some distance, touched the crural nerve with the point of a scalpel, “immediately all the muscles of the limbs seemed to be so contracted that they appeared to have fallen into violent tonic convulsions” (p. 23) (Fig. 1). This finding, however, was not the result of an experiment explicitly designed to produce or test this effect. While some commentators, such as Alibert (1801), uncharitably attributed this finding entirely to blind luck or chance, it is perhaps better understood as an instance of what Rheinberger (1997) calls *tâtonnement* (p. 45) or “organized groping,” wherein researchers pursue a variety of open-ended experimental avenues while remaining sensitive to the occurrence of “unprecedented events” which, if they persist, might prove worthy of further attention (p. 51–56). In fact, much of *De Viribus* proceeds in this manner, reflecting an experimental practice best understood as a series of “tinkered arrangements” built to support the “continuous reemergence of unexpected events” rather than decisive experiments designed to test hypotheses predicted by an established theory (Rheinberger, 1997, pp. 32–33). The element of surprise and recognition of the unexpected helps to underscore Galvani’s goals during this period, which were exploration, characterization, and probing, not explanation.

In another well-known experiment, Galvani demonstrated that lightning was sufficient to elicit muscle contraction when a wire connected to the nerve was suspended in his porch garden while another wire ran from the foot to a well. However, subsequent experiments based on this design soon led him to conclude that lightning was, in fact, unnecessary. Having noted that sometimes muscles with a bronze hook inserted into the nerve and laid on an iron grating on the porch would contract, Galvani reports that he

began to press the bronze hooks, whereby their spinal cords were fixed, against the iron gratings, to see whether by this kind of device they excited muscular contractions, and in various states of the atmosphere, and of electricity whatever variety and mutation they presented; not infrequently, indeed, I observed contractions, but bearing no relation to varied state of atmosphere or of electricity (p. 40).

Galvani was able to produce the same result using a variety of metals and under different conditions—for instance, placing the muscle in a closed chamber, isolated from the environment entirely. This, he says, “began to arouse some suspicion about inherent animal electricity itself” (p. 41).

**Fig. 2:**
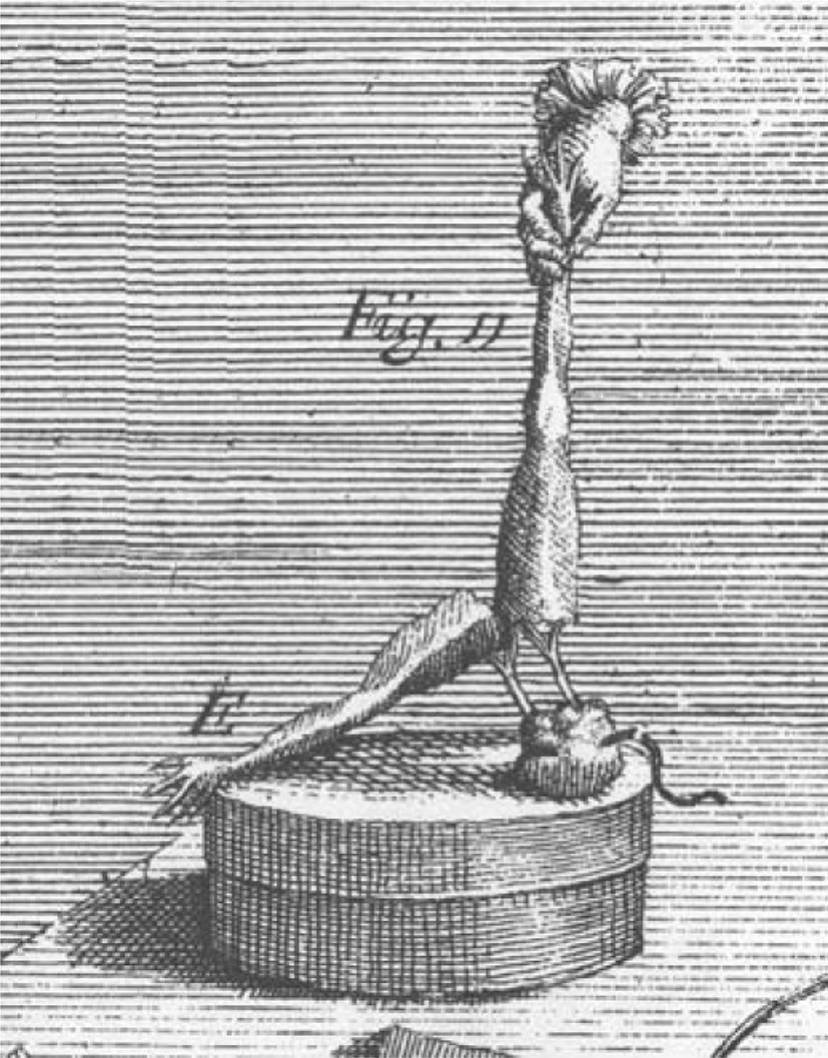
One leg of a frog held so that the hook in the nerve fell on a silver box. When the other leg fell onto the box, it would contract and raise up from the box. From Plate III in *De Viribus*

To investigate whether animals possess their own source of electricity, Galvani placed a prepared frog leg on an insulated surface and used a metal that would conduct electricity to connect the brass hook extending out from the interior to the exterior of the leg. This generated contractions. Among the most dramatic of these experiments, shown in Fig. 2, Galvani held one leg of a prepared frog over a silver box so that the hook in the nerve touched the box. Whenever the other leg fell on the box, the muscle would contract, breaking the contact until the leg fell again. The leg, he reported, acted “like an electric pendulum” (p. 44).

To make his proposal clear, Galvani advanced the analogy of “a muscle fibre to a small Leyden jar, or other similar electric body charged with the two opposite kinds of electricity." He elaborated by suggesting that the internal and external surfaces of the muscle correspond to the foil strips separated by glass in the Leyden jar. The nerve, on his account, “hooks” into the muscle just as the wire that protrudes from the stopper of the jar hooks into its interior. Crucially, both discharged electricity when the circuit between the interior and exterior was completed. Accordingly, Galvani likened the whole muscle to “an assemblage of Leyden jars.”

Alessandro Volta responded favorably to *De Viribus* at first; however, his enthusiasm was short-lived. He soon mounted a vigorous and sustained argument against Galvani’s claim that electricity originated in the frog specimen (Kipnis, 1987). Instead, he contended that the current arose from the contact between the different metals that Galvani claimed served to detect the current. Galvani had himself noted that if only one type of metal were used in his various experiments “the contractions will either fail, or will be very scanty” (p. 45). Taking two different metals as providing a source of electric current, Volta went on to create the electric pile or battery (Volta, 1800).

**Fig. 3:**
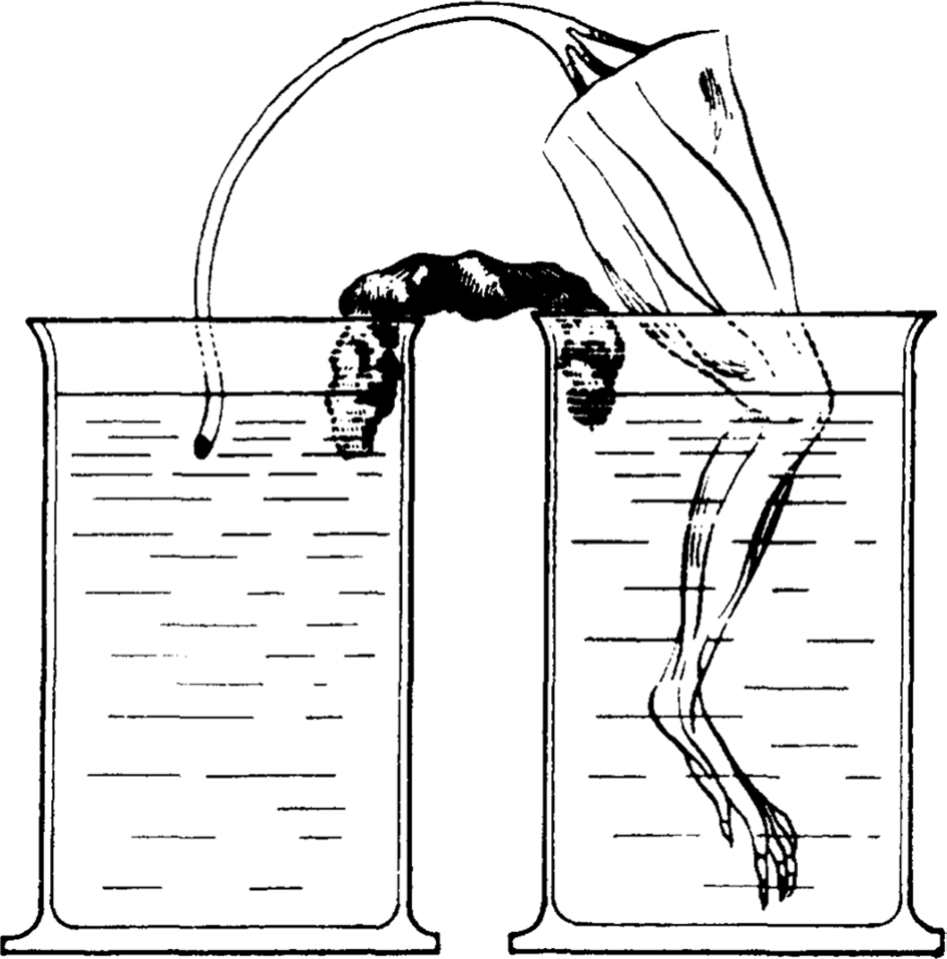
Galvani’s experiment showing that muscle contraction did not depend on metals—the circuit was closed by wet paper. From Sirol (1939)

Volta’s objections led many investigators to reject animal electricity over the following decades. In the remaining years of his life, however, Galvani, fought back, defending his claim that electricity arose in the muscle itself. He developed new experiments in which no metal was employed to make contact between the nerve and the exterior of the muscle (details of these experiments are related in Piccolino & Bresadola, 2013). In perhaps the most impressive demonstration, Galvani inserted the leg of the frog into one water-filled vessel and the nerve into another. Whenever he inserted moistened paper between the vessels, the leg would contract (Fig. 3).

Based on his investigations, Galvani concluded that electricity does originate in muscle and named the new phenomenon—*animal electricity*. It is significant that although Galvani offered numerous experimental demonstrations of the phenomenon, all involved using the frog muscle not just as the source of electricity but also as the instrument that detected it (by contracting). These two roles map neatly onto a distinction introduced by Rheinberger (1997, pp 28–29) between *epistemic things*—objects of scientific interest, targets for investigation—and *technical objects*—parts of the experimental conditions which support such investigations. There is not, however, a durable distinction between these two kinds of objects. Epistemic things often become technical objects once they have become “sufficiently stabilized,” but can likewise reemerge as epistemic things during the course of further research; the difference between the two, he contends, is “functional rather than structural” (pp. 29–30). That Galvani’s *frogs prepared in the usual manner* constitute a kind of hybrid, occupying both positions at once, helps illustrate just how flexible this distinction can be.

**Fig. 4:**
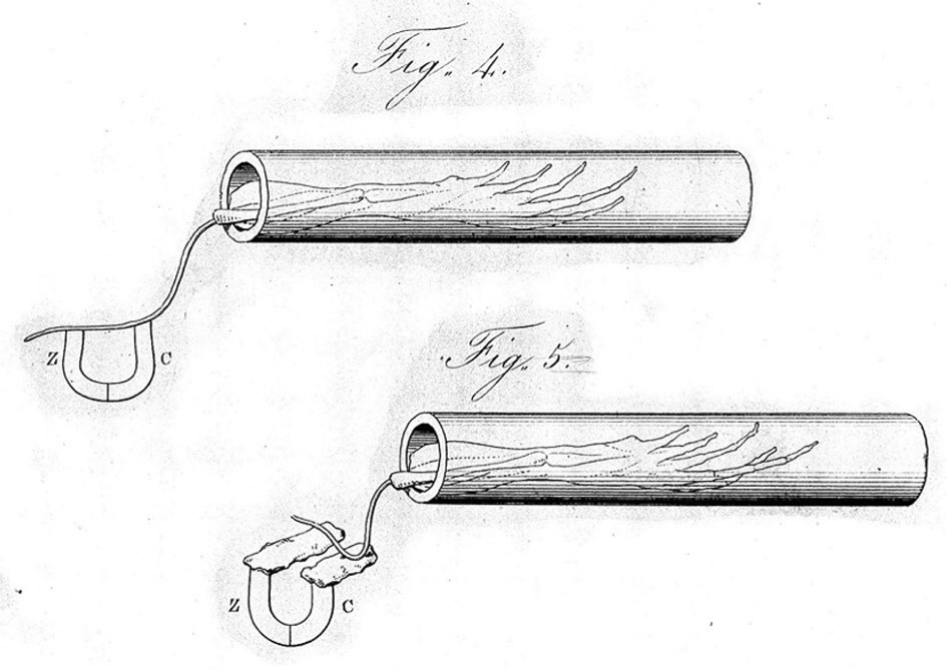
Matteucci’s galvanoscopic frog. From (Matteucci, 1844)

Subsequent researchers would continue to employ the frog for both roles. Matteucci referred to the prepared frogs as *galvanoscopic frogs*; du Bois-Reymond as *rheoscopic limbs*. As shown in Fig. 4, Matteucci situated the frog leg within a glass tube, with only the nerve extending out, so as to insulate it from other sources of electricity. Importantly, however, they developed alternative means of detecting what they took to be electrical current in muscle, thereby liberating the phenomenon from the sole experimental arrangement in which Galvani detected it. They also expanded on Galvani’s rather minimal characterization of the phenomenon provided by the analogy of a muscle fiber to a Leyden jar.

### A mechanical device for measuring electrical currents: the galvanometer

To characterize electrical currents in terms of strength, duration, and direction, subsequent researchers employed a new instrument, the galvanometer. Oersted (1820) identified the physical principle of electromagnetism on which the galvanometer relies when he observed that electricity flowing through a wire could swing a nearby magnetized needle. Schweigger (1821) demonstrated that by coiling the wire and situating a magnetized needle within the coil, he could detect an even weaker current. Schweigger referred to his instrument as a *multiplikator* but also, after Galvani, as a *galvanometer*. But the galvanometer could do more than detect currents. After initial oscillation, the needle would hold its position as long as the current flowed, with its position providing a measure of strength. Moreover, it could turn in either direction, indicating the direction of the current.^5^

**Fig. 5:**
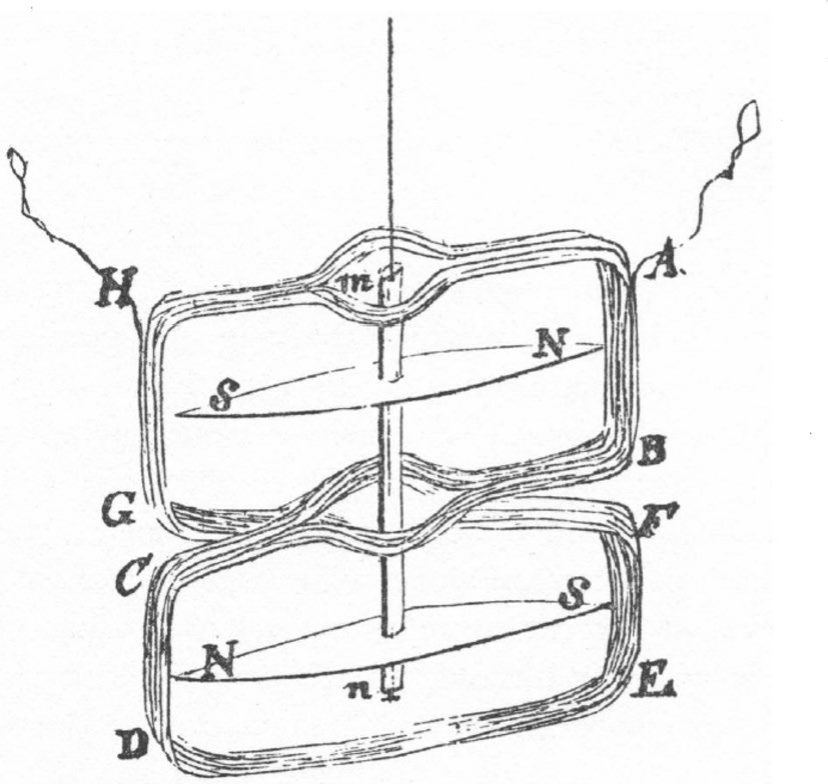
Illustration of Nobili’s wiring for his galvanometer, appended to Nobili (1825) by Schweigger, who was the editor of the journal

Neither Oersted nor Schweigger applied the galvanometer to animal tissue. Leopold Nobili took up this project. To detect the weak currents in animal tissue, Nobili (1825) wound the wire 72 times. As a result, his galvanometer was also sensitive to the magnetic field of the earth, leading him to develop a design in which wire was wound in a Fig. 8 (Fig. 5). Nobili inserted a magnetized needle in each loop and connected them so that they moved together. Each needle would be affected by the earth’s magnetism in the opposite direction, but since the needles were connected, these effects would cancel out. As a result, the galvanometer would only register the current passing through the wire.

Nobili (1828) inserted his galvanometer into the design of Galvani’s experiment illustrated in Fig. 3 and demonstrated the existence of a current which he labeled *courant de grénouille* or *courant propre* (frog current or proper current). Next, he tested the effects of putting multiple frogs into a circuit and found that if the nerve of one contacted the muscle of the other, both would contract; however, if he connected nerve to nerve and muscle to muscle, there would be no contraction. Nobili interpreted this as showing that in the latter arrangement, the two currents canceled each other. Building on these findings, he arranged multiple muscles into a circuit—taking care to only connect muscle to nerve—and showed that the current detected with the galvanometer would increase with each additional muscle. Nobili did not attempt to determine which component, muscle or nerve, was responsible for the current, but simply attributed the current to the whole preparation.

### Detecting currents in isolated muscles (and nerves)

**Fig. 6:**
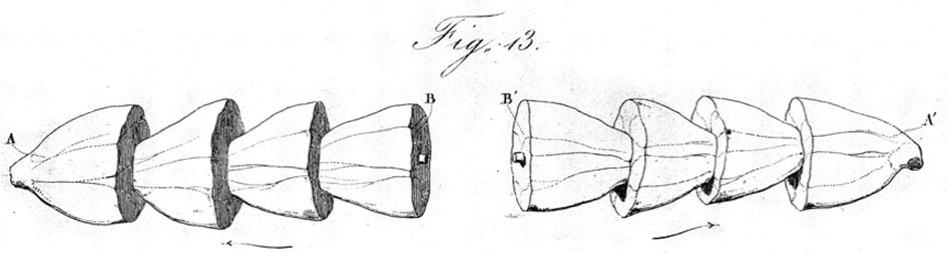
Matteucci’s frog pile. The arrows indicate the direction of current flow. From (Matteucci, 1844)

Following upon Nobili’s work, Matteucci (1838, 1840) constructed his own galvanometer, adding additional windings to better detect weak currents. In his first studies, he placed the leg of a prepared frog in one vesicle and the muscle and attached nerve in another and connected each to a different pole of the galvanometer. When he connected the two with wet cotton, he reported that the galvanometer registered “a very perceptible deviation, always directed in the same direction” (1838, p. 99). Matteucci referred to this as the *proper current*. In his subsequent investigations he wounded muscle in a living animal (pigeon, rabbit, or ewe) and determined he could record a current in it by “plunging one of the electrodes into the wound and resting the other on the exposed surface of the injured muscle.” He reported that the “current was directed from the inside of the wound to the outside surface of the muscle” (Matteucci, 1842, p. 331). The current from one muscle was extremely weak, but Matteucci followed up on Nobili’s proposal of arranging several muscles so that the uncut surface of one touched the cut surface of the next (an arrangement he referred to as a frog pile). He found that the current increased in proportion to the number of muscles included (Fig. 6).^6^

Emil du Bois-Reymond^7^ began his large-scale investigation into electrical currents in both muscles and nerves after his mentor, Müller, passed on a copy of Matteucci’s monograph (Matteucci, 1844) and suggested that he might find in it a possible topic for research. In preparation for this undertaking, du Bois-Reymond constructed his own, much more sensitive, galvanometers. One, which he used with muscles, wound 3280 feet of wire 4,650 times while another, which he used with nerves, wound 3.17 miles of wire 24,160 times.

**Fig. 7:**
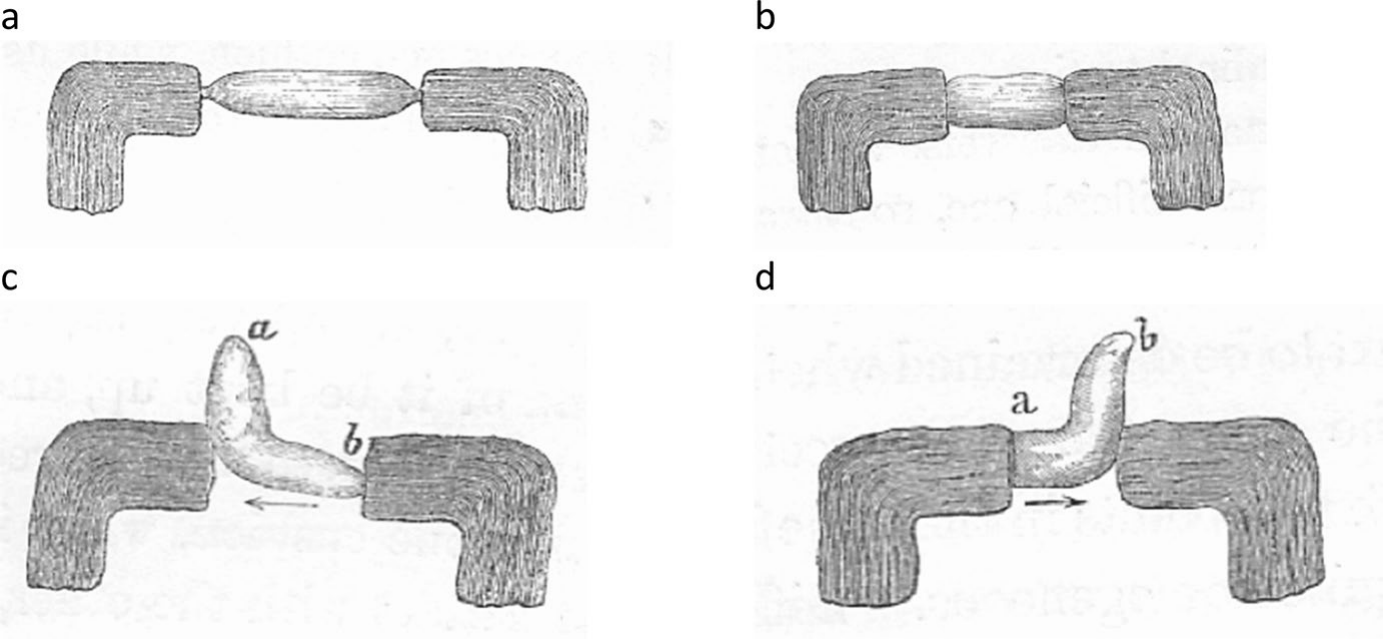
Different arrangements of a muscle on the pads of a galvanometer. The arrangements shown in c and d produced a current, as indicated by an arrow. From (du Bois-Reymond, 1852)

So equipped, du Bois-Reymond systematically varied the position of the muscles and nerves on the pads of the galvanometers, recording the effects on the current that was detected.^8^ No current was detected if he either touched one tendon of a muscle to each pad (Fig. 7a) or cut the muscle transversely, placing one cut end on each pad (Fig. 7b). However, if the longitudinal surface was placed on one pad and either the tendon (Fig. 7c) or transverse cuts through the muscle (Fig. 7d) on the other, a current would be detected by the galvanometer. Moreover, as indicated by the arrows, the current would flow from the tendon or transverse cut to the longitudinal surface. From this du Bois-Reymond arrived at the conclusion that “Every point in the natural or artificial longitudinal section of a muscle is positive in relation to the transverse section whether natural or artificial.” He went on to show that current could also be procured if a point nearer the center of the muscle on either the longitudinal or transverse surface were placed on one pad and a point further from the middle were placed on the other. In comparing what he termed the *electromotive force* of the whole muscle with longitudinal or transverse slices through it, he concluded that the slices generated a greater electrical current. Du Bois-Reymond also engaged in comparative examination of different muscles and concluded that the “electromotive force of muscles increases both with their length and thickness” (du Bois-Reymond, 1852, p. 115).

**Fig. 8:**
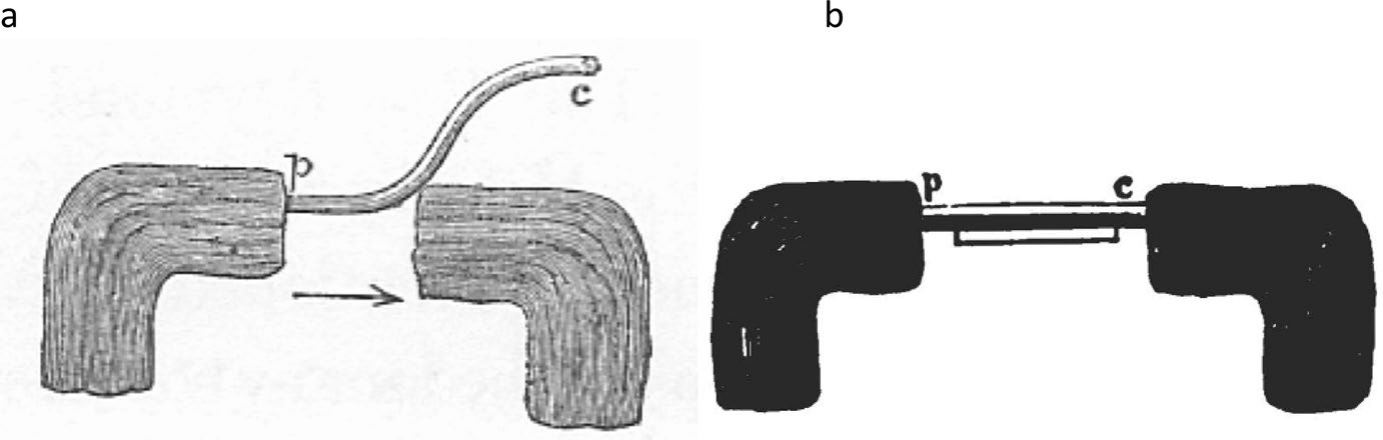
Ischiatic nerve placed on the pads of a galvanometer. From (du Bois-Reymond, 1852)

1. Du Bois-Reymond carried out similar studies in nerves. Although the current was much weaker (requiring the galvanometer with many more windings), du Bois-Reymond found that if he placed the end produced by a transverse cut of the ischiatic nerve on one pad and a longitudinal section on the other, he could detect a current (which he referred to as the *nervous current*), again flowing from the transverse cut to the longitudinal surface (Fig. 8a). No current was detected if transverse sections abutted the pads on both ends (Fig. 8b). Subsequent comparisons between sensory and motor nerves revealed no differences between them: “The electromotive action of the anterior and posterior roots, does not present any perceptible difference. The transverse section is negative in regard to the longitudinal section in the motor and sensory, as well as in the mixed nerves” (p. 169). He concluded that an electrical current was intrinsic to both muscles and nerves.

Ostensibly, the muscle and nerve current reported by du Bois-Reymond was the same as the animal electricity reported by Galvani and detected with a galvanometer by Nobili and Matteucci. For all of them, there is a current in muscle or nerve tissue that could be detected with either the galvanoscopic frog or the galvanometer. And yet, the phenomenon is not the same. Rather, the intuitive appeal of treating these early findings as instances of “the same thing” helps to underscore a dangerous misconception that Rheinberger (1997) flags when he notes that once an unexpected finding has been “sufficiently stabilized, it becomes more and more difficult…to avoid the illusion that it is the inevitable product of logical inquiry or of a teleology of the experimental process” (p. 74). However, careful scrutiny of the details here helps illustrate why this was not the case. For one, Nobili, Matteucci and du Bois-Reymond introduced different names for what Galvani had referred to simply as “animal electricity.” In some instances, giving the phenomenon a new name served to limit its scope, as was the case with Nobili’s *frog current*, or render it more specific, as with du Bois-Reymond’s *nervous* and *muscular* currents. Unsurprisingly, these new terms often carried with them new interpretations of the phenomenon. Nobili, for instance, mistakenly believed the current to be a thermoelectric phenomenon which resulted from temperature differences between muscle and nerve due to evaporation (Moruzzi, 1996). Finally, with the introduction of the galvanometer and specific procedures for employing it, researchers were able to measure and describe new aspects of the phenomenon such as the direction of flow and the strength of the current. At this stage, however, the reports of what was detected remained limited to the experimental procedures and conceptual framings of the different investigators. As such, it was far from obvious at the time whether a single, coherent characterization of the phenomenon would emerge from the variety of options on offer.

### Dismissing the muscle and nerve currents as artifacts of injury

Up to this point, du Bois-Reymond’s work on animal electricity represented the most detailed and systematic work on the topic available; however, like Galvani, his work soon became the focus of a critical challenge, which suggested the nervous and muscular currents were in fact experimental artifacts. As we noted above, Matteucci reported that he injured muscles in the course of recording from them. Du Bois-Reymond denied this was of any significance, insisting that the current existed in the muscle prior to injury. To defend this claim, he applied a saturated salt solution to the skin of a living frog and measured the current with his galvanometer. The results, he argued, showed that “By this management the skin is changed into a perfectly inactive moist conductor, and the current of the frog, as discovered by Nobili, may be observed on the live and unhurt animal” (p. 126). While he conceded that the current was weaker than when measured in isolated muscle, he attributed this difference to the presence of skin. His reasoning seems to have been that since the frog was still alive, it was not injured by the treatment of the skin.

It was one of du Bois-Reymond’s own students, Ludimar Hermann, who—following up on the clue in Matteucci's report—began to investigate whether the muscular current was an artifact of injuring the muscle during preparation. By minimizing the injury he inflicted prior to measurement, he found that the current was noticeably reduced: “the more precautions I took to avoid all damage, the weaker the currents became, so that I was finally forced to the conviction that even exposed gastrocnemii are currentless when all damage is avoided; exposure itself can have, at most, an extremely weak effect” (Hermann, 1870, p. 35).^9^ Accordingly, Hermann referred to the current measured in prepared muscles as the *injury current* or *demarcation current*; by the latter term he referenced the finding that the current only appears at the demarcation between normal and injured tissue.

**Fig. 9:**
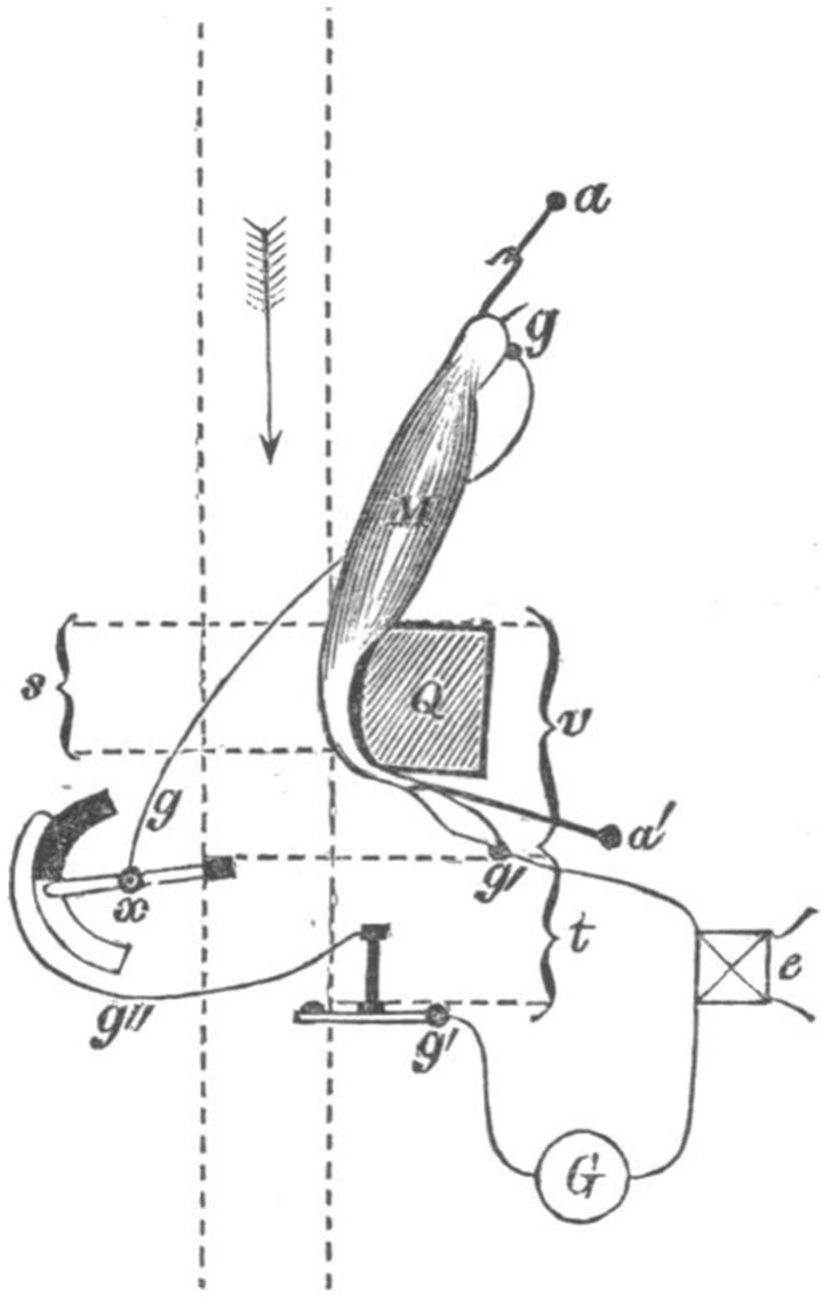
Hermann’s fall rheotome as diagrammed by Burdon-Sanderson (1878). After first injuring the muscle M, the falling block moves the lever *x* (closing the circuit *g-g’’*) and then a second lever, *g’*, which reopens the circuit

To demonstrate how current increased in the period following injury, Hermann adapted the rheotome, “an instrument which periodically interrupts a current” (Wheatstone, 1843; for a history of rheotomes, see Hoff & Geddes, 1957), so that the circuit connecting the muscle to the galvanometer would be opened during a limited time window after the muscle was injured. He called his rheotome a *fall* rheotome since a falling block would first injure the muscle and then, during a subsequent interval, close and then re-open the circuit to the galvanometer (Fig. 9). The distance between the muscle and the levers was adjustable, allowing the operator to measure how long after the impact on the muscle the galvanometer registered the current. If the muscle had not previously been injured, the current would increase as the distance between the muscle and the recording levers was increased. However, if it had been previously injured, it would exhibit the maximum, regardless of how distant the muscle was from the levers. In reporting this study for an English-speaking audience, the prominent British physiologist Burdon-Sanderson (1878) quoted Hermann: “It is proved that the [current when the muscle was not previously injured] is not immediate, and, consequently, that the electro-motive forces of which it is the manifestation are not in operation at the moment that the cross surfaces of the muscular fibres are exposed.”

One could view Hermann’s demonstration that the muscle current only arose after injury in one of two ways, either showing that the phenomenon Galvani reported as animal electricity and du Bois-Reymond as the muscle current was simply an experimental artifact, or that it was a real but previously mischaracterized phenomenon. Hermann adopted the latter strategy. He introduced a new name—the *injury current*—and characterized it in sharply different terms than his former teacher. Hermann’s choice is significant for at least two reasons. First, it indicates that despite rejecting du Bois-Reymond’s conception of the phenomenon, he remains convinced that there is *some* phenomenon worthy of investigation. Subtle as it may be, this is a milestone in the lifecycle of a phenomenon; experimenters may disagree about exactly how to describe it, but coming to recognize that there is something to describe (above and beyond the unintended effects of their instruments and interventions) is a genuine development. Second, it shows how, by employing additional instruments (the fall rheotome) and alternative conceptualizations (linking the current to injury) a phenomenon is extracted from the specific context in which it was first established. Moreover, Hermann is able to retain most of what had been attributed to muscle and nerve currents by previous investigators with the proviso that the current only arose in the injured muscle or nerve.

### What pre-exists: not a current but a potential

The conflict between du Bois-Reymond and Hermann was heated, with du Bois-Reymond not only expelling Hermann from his laboratory but vociferously challenging his experimental competence in print (du Bois-Reymond, 1877). Du Bois-Reymond’s contention that the current pre-existed injury to muscle came to be referred to as the *preexistence theory*. Another researcher in du Bois-Reymond’s laboratory, Bernstein (who was also childhood friends with Hermann), became this theory’s primary champion (for details on Bernstein, see Seyfarth, 2006). But he abandoned the idea that a current preexisted. Rather, according to Bernstein (1894) what preexisted was voltage: du Bois-Reymond’s “theory of muscle and nerve currents presupposed the pre-existence of electrical voltages in the molecules of the fiber.” Soon after, Bernstein (1902, 1912) developed an account of voltage in terms of a potential difference across the membrane that could, once the membrane was breached, result in a current.

To develop his account of the membrane potential, Bernstein drew upon a different body of research that began with Traube’s (1867) demonstration of the phenomenon of osmosis: liquids pass through a semi-permeable copper ferrocyanide membrane so as to equalize the concentration of solutes that cannot pass from one side to the other. For Traube osmosis provided a mechanical model of cell growth. van’t Hoff (1887) extended the account of osmosis to charged particles—ions—that result from the dissociation after crystalline salts are dissolved in water. Building on this work, Wilhelm Ostwald, proposed that when one ion, but not the other, can freely cross the membrane, an electrical charge is established:

. . . the positive ions are not able to cross the membrane, but the negative ones can. Then, by virtue of their osmotic pressure, the latter will soon pass through the membrane. But this results in a separation of the electricity, and the resulting electrostatic forces, when they have become equal to the osmotic pressure of the negative ions, prevent them from further passage. This results in an electrical double layer on the membrane, the positive side of which, in this case, is on the solution side and the negative side is on the water side. (Ostwald, 1890, p. 73).

He went on to propose “It is perhaps not too bold to suggest that not only the currents in muscles and nerves, but also especially the enigmatic effects of the electric fish, will be explained by the properties of the semipermeable membranes discussed here” (Ostwald, 1890, p. 80). Katz (1896) supported Ostwald’s proposal with measurements of the concentrations of different cations in various types of muscles and the surrounding plasma, demonstrating that K^+^ is in much higher concentration inside muscles than in the plasma while the concentrations of Na^+^ is higher outside.

When ions are at different concentrations across the membrane and the membrane is breeched, the flow of ions registers as a current. While the membrane is intact, there is no current, but a potential, measured as a voltage. Nernst (1889, p. 162) introduced an equation for determining the voltage that provided a “decomposition of the electromotive force into its component concentrations.” Represented in contemporary terms, the equation relates the voltage (*V*) to the concentrations of an ion inside ([*ion*]_*in*_) and outside ([*ion*]_*out*_) the cell membrane:

$$V = \frac{RT}{{Fz}}\ln \frac{{ion_{in} }}{{ion_{{{\text{out}}}} }}.$$

In this equation, R is the gas constant, *T* absolute temperature, *F* the Faraday constant, and *z* the valence of the ion.^10^

**Fig. 10:**
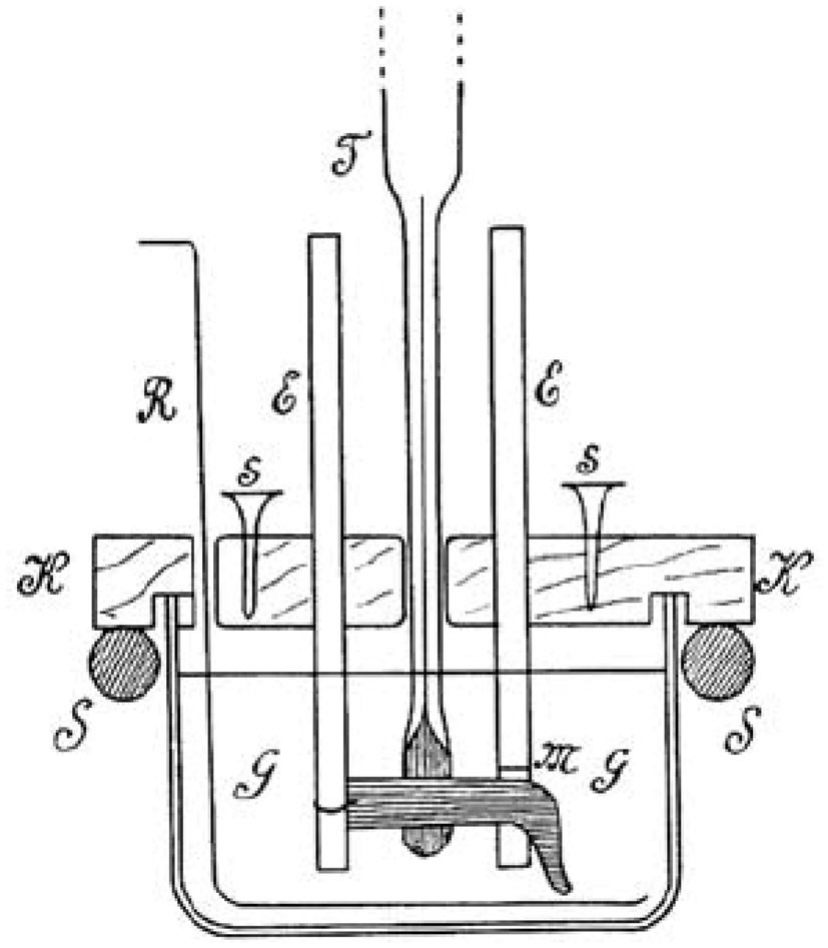
Bernstein’s (1902) apparatus for simultaneously measuring temperature and injury current. Electrodes E contact respectively the transverse section and the longitudinal surface of muscle (M) in oil in a glass jar (G) sealed by a cork lid (K). A thermometer (T) registers the temperature on the muscle surface while a stirrer (R) ensures that the oil through the jar is at the same temperature

At least by 1900 Bernstein knew of this research as he added a new appendix to his *Lehrbuch* (1900) describing it and citing among other sources, Nernst’s *Theoretische Chemie* (Nernst, 1893*)*. Two years later, Bernstein (1902) made the Nernst equation the foundation of a revised characterization of what preexisted the injury currents detected in muscles and nerves: an electrical potential generated by an unequal distribution of K^+^ ions.^11^ To support this hypothesis, Bernstein drew upon research he was already doing on the energy requirements of muscle contraction that focused on the effects of temperature (Bernstein & Tschermak, 1902). According to the Nernst equation, the resting/injury current should increase linearly with increase in temperature. Bernstein created an apparatus in which he could simultaneously measure the temperature and the injury current produced by a muscle cut transversely (Fig. 10). Recording the current for various temperatures between -2° C. and + 36° C., he determined that the strength of the current increased linearly with temperature (the same holding true for nerves between 9 °C. and 18 °C.).

After finding that the current generated by breaching the muscle membrane increased linearly with temperature—which the Nernst equation predicted if there were unequal distributions of ions across the membrane—Bernstein proposed that the charge (potential) over the membrane corresponded to the unequal distribution of K^+^ across the membrane:

Let us imagine that these electrolytes diffuse unhindered from the transverse section of the fibrils into the surrounding fluid, while at the longitudinal section their diffusion is inhibited by the living sarcoplasmatic membrane. For example, if the anion (PO_4_^-^, etc.) is more or less impermeable, then a double electric layer will form on the surface of the fibril, with a negative charge on the inside and a positive charge on the outside (Bernstein, 1902, p. 542).

He concluded: “the electrical potential of the lesioned muscle is caused by the electrolytes, in particular by inorganic salts such as KH_2_PO_4_, already contained in the undamaged muscle fiber” (p. 541–542).

**Fig. 11:**
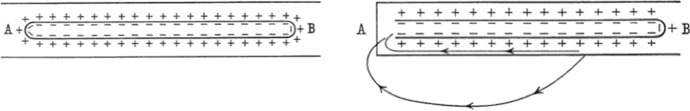
Left. Bernstein (1912) proposal of a negative charged layer inside the membrane and a positive charged layer outside the membrane. Right. When the cell is transected, a current results which can be detected flowing from the outside of the membrane to where the cell is transected

Bernstein’s efforts transformed how researchers understood the phenomenon, completing a process that began with Galvani and continued through to Hermann. He denied a preexisting current in either muscle or nerve—the actual current only arose with injury. What preexisted was what he characterized as a double layer of positive and negative charges across the cell membrane (Fig. 11A). When the cell was injured by a transverse cut, a current would flow from the more positive exterior to the negative interior Fig. 11B).

### Reflections on the discovery of the membrane potential

The history of research culminating in Bernstein’s demonstration of the membrane potential shows how appropriate experimental procedures, findings, and concepts, developed over time, permitted the extraction of the phenomenon from particular (and isolated) experimental contexts, eventually leading to its characterization as a natural process. Galvani’s research revealed that something electrical happens in muscles and nerves. Doing so required a detection device—Galvani used a prepared frog muscle that, by its contractions, registered the presence (actually, the onset or termination) of a current. He used the Leyden jar as a model for what was present in muscle. The path from this to the recognition of the membrane potential played out over a century with the introduction of new instruments, especially the galvanometer and the fall rheotome, new findings, and the introduction of new concepts. Du Bois-Reymond played a central role, not only confirming the existence of the current Galvani reported but providing a much more detailed description of it (e.g., establishing that it flowed from transverse sections to longitudinal sections of muscles and nerves).

The process was not just one of accumulating new findings. Matteucci had recognized that the current was measured in injured muscle and Hermann demonstrated with the fall rheotome that the current grew over the period following injury, indicating that no current existed in the uninjured muscle. Accordingly, he renamed the current observed by Galvani through du Bois-Reymond the *injury current*. The introduction of new tools and protocols meant new sources of data, which in turn opened the door to more elaborated characterizations of the phenomenon. However, these same forces also pulled researchers in different directions, leading to a proliferation of new concepts, many of which were inconsistent with those advanced earlier. While Hermann challenged du Bois-Reymond’s claim to a preexisting current, Bernstein, held on to preexistence, but changed the conception of what preexisted to a potential resulting from an unequal distribution of ions across the membrane. Through the process of developing new experimental strategies for gaining information about the phenomena and sometimes competing conceptualizations, a century of inquiry arrived at a conception of the membrane potential that generalized beyond specific experimental designs and was projected as a general feature of muscles and nerves.

At the outset, we noted a tension between two influential conceptions of scientific phenomena found in the literature: Hacking’s view which takes phenomena to be the products of highly specific laboratory arrangements (i.e., events which occur rarely and only under special conditions), and Bogen and Woodward’s position which situates phenomena as part of the natural order. The history recounted above provides an initial sketch of how these two views might begin to be reconciled by illustrating how phenomena come to be “recursively constituted,” to borrow a phrase from Rheinberger (1997, p. 76). Indeed, at the heart of this process lies a familiar opposition. On the one hand, experimental systems, according to Rheinberger, are fundamentally “localized and situated,” which is to say that are in some sense, isolated from one another (p. 76). Accordingly, phenomena (or scientific objects more generally) are contained within the technical conditions of such systems in both senses of the word — the conditions “embed them” as well as “restrict and constrain them” (Rheinberger, 1997, p. 29). On the other hand, establishing a phenomenon requires that it be “inserted into the reproductive cycle of an experimental system” (Rheinberger, 1997, p. 76). The reproduction of an experimental system is, for Rheinberger, a dynamic affair, wherein the material conditions of experimental inquiry evolve over time, both sustaining the possibility for continued investigation while simultaneously driving change over time. Taken together, this suggests that scientific objects are both contained within isolated experimental contexts and yet, in order to become established, must in some sense transcend these boundaries and undergo a continual process of re-definition by becoming embedded in related, but nonetheless distinct experimental setups.

## From a curious termination of current to the action potential

A natural assumption, embraced by Galvani and others, was that electricity flowed through nerves much like water in pipes. This fit the then accepted conception of electricity as an imponderable fluid. The idea of an action potential, however, offers a very different characterization—what is passed along a muscle or nerve is a reduction or reversal of the membrane potential. This framing, articulated by Bernstein, only makes sense once the idea of a preexisting muscle current is replaced with the idea of a membrane potential. But the action potential, as a reversal of the muscle current, made its appearance and was described in some detail well before that.

### First detection

What we now recognize as the action potential was initially reported in Matteucci’s (1838) first paper on the proper current. In it, Matteucci noted that when he subjected a muscle to a barrage of stimulations until it would no longer respond (inducing what is known as the tetanic state), the proper current would disappear:

another factor that greatly modifies the frog’s proper current is its tetanic state. It often happens that in quickly preparing active specimens we see them extend and stiffen their legs to such an extent that it is impossible to bend them. . . . The influence of tetanus is such that the proper current is lacking while the frog is in it. We have no more contractions, nor signs with the galvanometer. If the animal is killed by poison, the current does not reappear, but if the tetanus was produced by the stimulation given to the frog while preparing it, once the convulsions have passed, the signs of the proper current reappear (Matteucci, 1838, pp. 102-103).

This reduction in the proper current was, for Matteucci, of secondary interest. His focus remained on the proper current itself. In his further research Matteucci continued to report on the effects of tetanus, often asserting that the proper current was not eliminated but only reduced in tetanus. For Matteucci, tetanus interrupted the production of the intrinsic current—it did not itself represent a feature of normal muscle activity.^12^

This case, perhaps even more clearly than Galvani’s, provides another example of a scientific object which began its career as a “boundary phenomen[on]” or noise (Rheinberger, 1997, p. 21). The contractions in Galvani’s frogs during the operation of the electrostatic machine were unexpected, but also an immediate source of interest. Matteucci, however, after initially finding the reduction in current during tetanus to be of interest, subsequently rejected it. du Bois-Reymond, on the other hand, not only accepted Matteucci’s finding of a decrease in the proper current but attributed great significance to it—he came to see it as the signal that was transmitted in nerve and muscle, responsible for eliciting contractions. He began his investigations by confirming Matteucci’s observation of reduced current during tetanus: “during violent and prolonged contractions the current is far from disappearing, but noticeably diminishes in intensity” (du Bois-Reymond, 1843, p. 12). But his focus was not on tetanus per se, but on muscle contraction; tetanus was merely a vehicle to reveal events that happened during muscle contraction which could not be observed directly in individual contractions due to the slow response of the galvanometer. To induce tetanus, he devised an instrument using a rotating disc to repeatedly administer an electric shock, each causing a contraction of the muscle. Repeated shocks induced convulsion. Once the muscle was in a tetanic state, du Bois-Reymond reported that “the needle is deflected through the zero point, and is seen to oscillate on the negative side of the zero, until the contracting power of the muscle is exhausted, which always happens before the needle has had time to come to rest.” (du Bois-Reymond, 1852, p. 132). du Bois-Reymond referred to this as the *negative variation*.

**Fig. 12:**
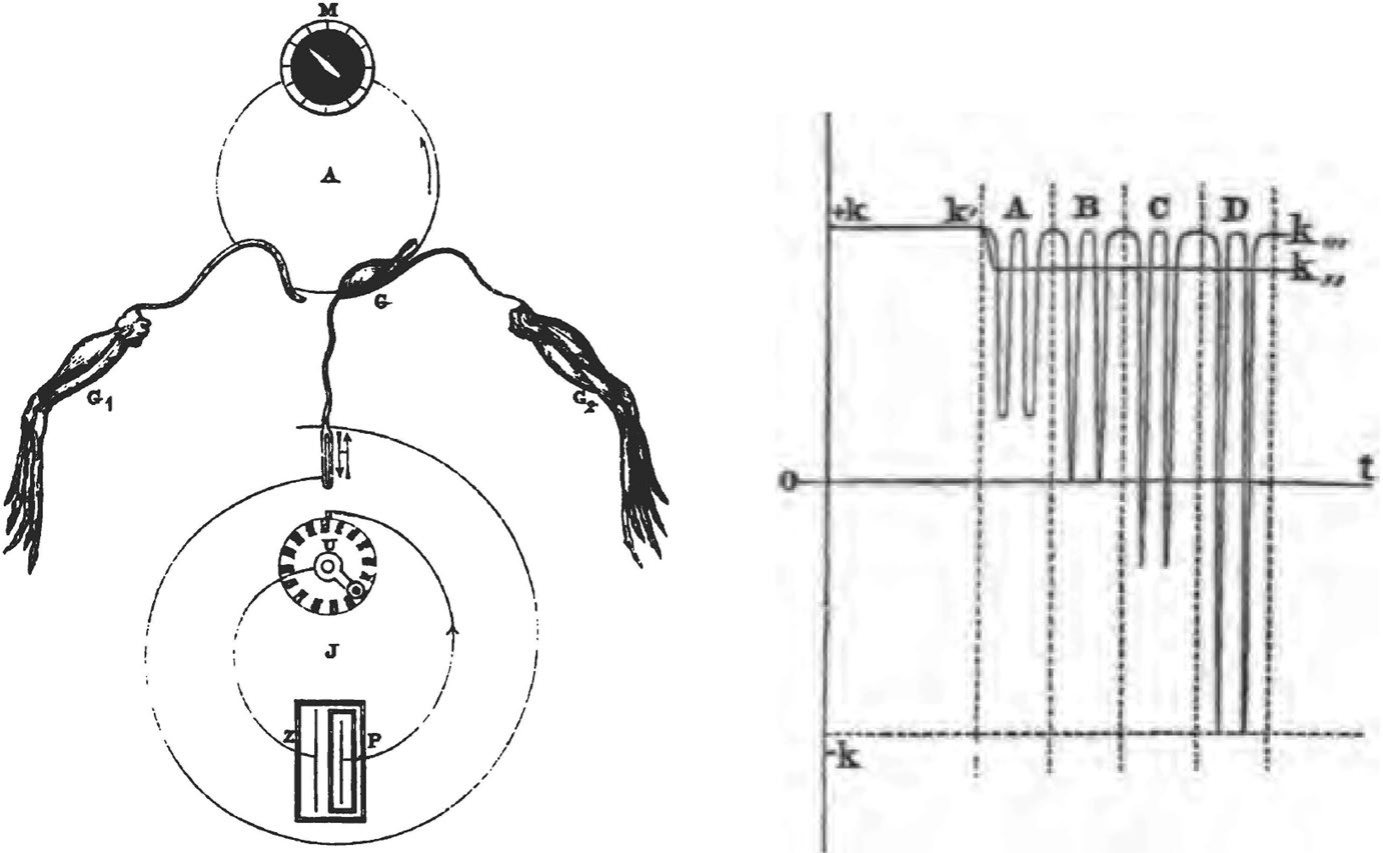
**A** Experimental setup in which a sequence of stimulations was administered to the nerve attached to one frog leg and a galvanometer and rheoscopic limb completed the circuit. **B** Hypothesized possible comb-like negative variations of the muscle current during tetanus, ranging from modest reduction, to elimination of all current, to a reversal of the current. From du Bois-Reymond (1852)

In order to demonstrate that the negative variation was a response to each stimulation of the muscle, du Bois-Reymond returned to the rheoscopic limb, which would contract immediately in response to each change in current. As shown in Fig. 12A, he inserted the rheoscopic limb into the circuit along with the galvanometer so that both responded to the repeated stimulations generated by the rotating disc shown at the bottom.^13^ He found that as the first muscle was being tetanized, so too was the rheoscopic limb. Since the rheoscopic limb only contracted in response to changes in current, he reasoned that for it to enter the tetanic state, it had to be receiving a sequence of stimulations. Accordingly, du Bois-Reymond (1850a) interpreted the response of the rheoscopic limb as an indication of successive negative variations in the first muscle and in Fig. 12B proposed four possibilities (varying in severality of the negative variation) of what happens in tetanus: “If time is represented on the abscissa and the intensity of the muscular current at each instant on the ordinate the, the curve does not exhibit a continuous inflection during tetanus, but takes the shape of a comb.”

du Bois-Reymond (1850b) also demonstrated the change in current in humans when they simulated tetanus by maintaining their muscles in a contracted state. To do this, he had a person (initially himself) dip fingers from both hands into salt water connected to the poles of a galvanometer. He would instruct the person to first sit quietly. The galvanometer would exhibit some deflections but settle on 0, which du Bois-Reymond interpreted as reflecting that the current in one arm cancelled that in the other. He then had the person contract the muscles in one arm while not actually moving it. He reported the needle deflected immediately, registering a current from the hand to the shoulder: “It may be concluded from this experiment that the muscular current in the arm, *when in a state of rest*, is directed from the shoulder to the hand, or is a downward one; and that the muscular current of the arm in the act of contraction, undergoes the negative variation, whereby the current in the other arm becomes the stronger, the result of which must be an apparent upward current in the contracted arm” (du Bois-Reymond, 1852, pp. 153–154).

**Fig. 13:**
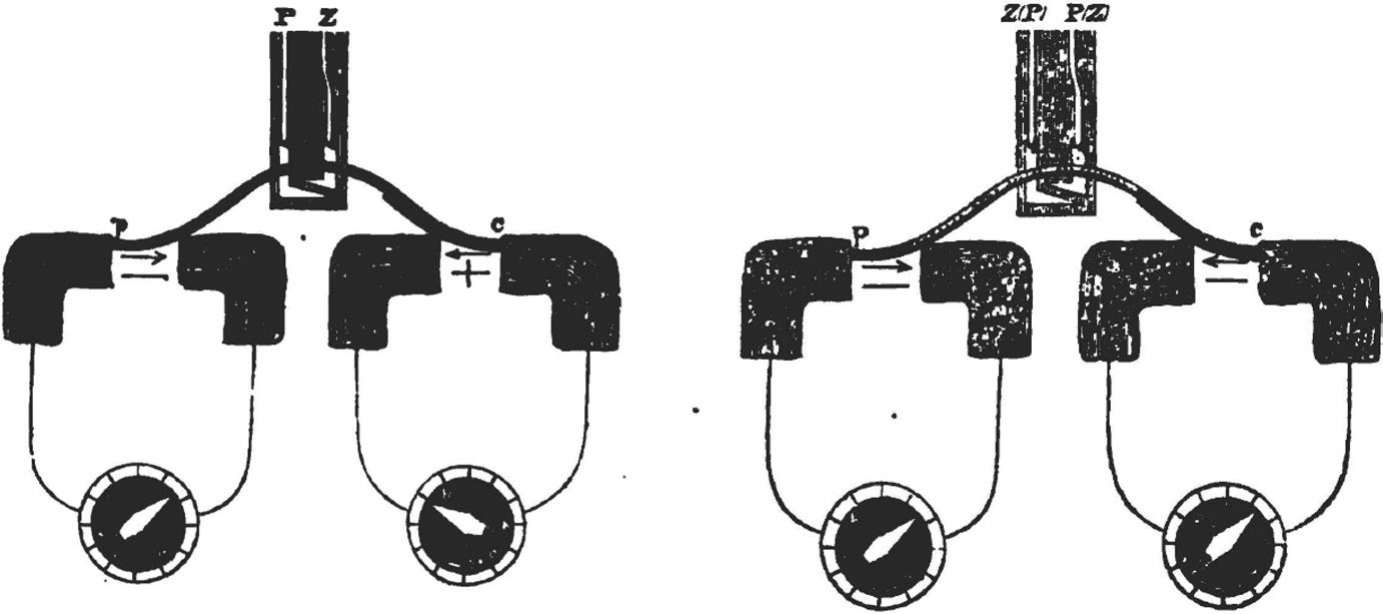
*A* Electrotonic state of nerve due to current from the battery shown at the top. **B** Negative variation resulting from alternating the current. From du Bois-Reymond (1852)

The conclusion du Bois-Reymond drew from these experiments was that the negative variation is a general phenomenon that occurs during each muscle contraction and is a fundamental part of it. Having established a current in nerves as well as muscle, du Bois-Reymond investigated whether it too would exhibit a negative variation. To do this, he created a setup with two galvanometers, connecting one end of an isolated nerve to the opposing pads of the two galvanometers and laying the intervening section over the other pads. Normally each galvanometer would record the nervous current in the direction indicated by the arrow; in this arrangement, they would then cancel each other out. He then made contact between the middle section of the nerve and the two poles of battery. As shown in Fig. 13A, this served to register a positive current on one galvanometer and a negative current on the other. Du Bois-Reymond described the situation thus: “When any portion of the length of a nerve is traversed by an electric current, beside the usual electromotive action of the nerve, a new electromotive action takes place in every point of the nerve, which has the same direction as the exciting current itself” (p. 180). He referred to this state of the nerve as the *electrotonic state.*

Du Bois-Reymond then used this setup to investigate the negative variation in nerve. Rather than a simple battery, he employed the interrupting wheel to repeatedly make and break the circuit. The current resulting from completing the circuit would be opposite to that when breaking it, resulting in an alternation. In this condition, du Bois-Reymond reported “the needle goes back to the zero point, and in favourable circumstances is deflected to the negative side” (p. 189) (see Fig. 13B). The connection between the negative variation and electrically induced nerve tetanus suggested to du Bois-Reymond that the former involved a change in the nerve that would generate a contraction upon reaching muscle: “The negative variation of the current, therefore, denotes a decrease in the electromotive force of the nerve when tetanised; and it may with great probability be considered as being in some way intimately related to that molecular change in the interior of the nerve, which, when it reaches the muscle, will produce contraction, or when it reaches the brain will be perceived as sensation.” (pp. 191–192).

### Identifying the negative variation with the nerve current

**Fig. 14:**
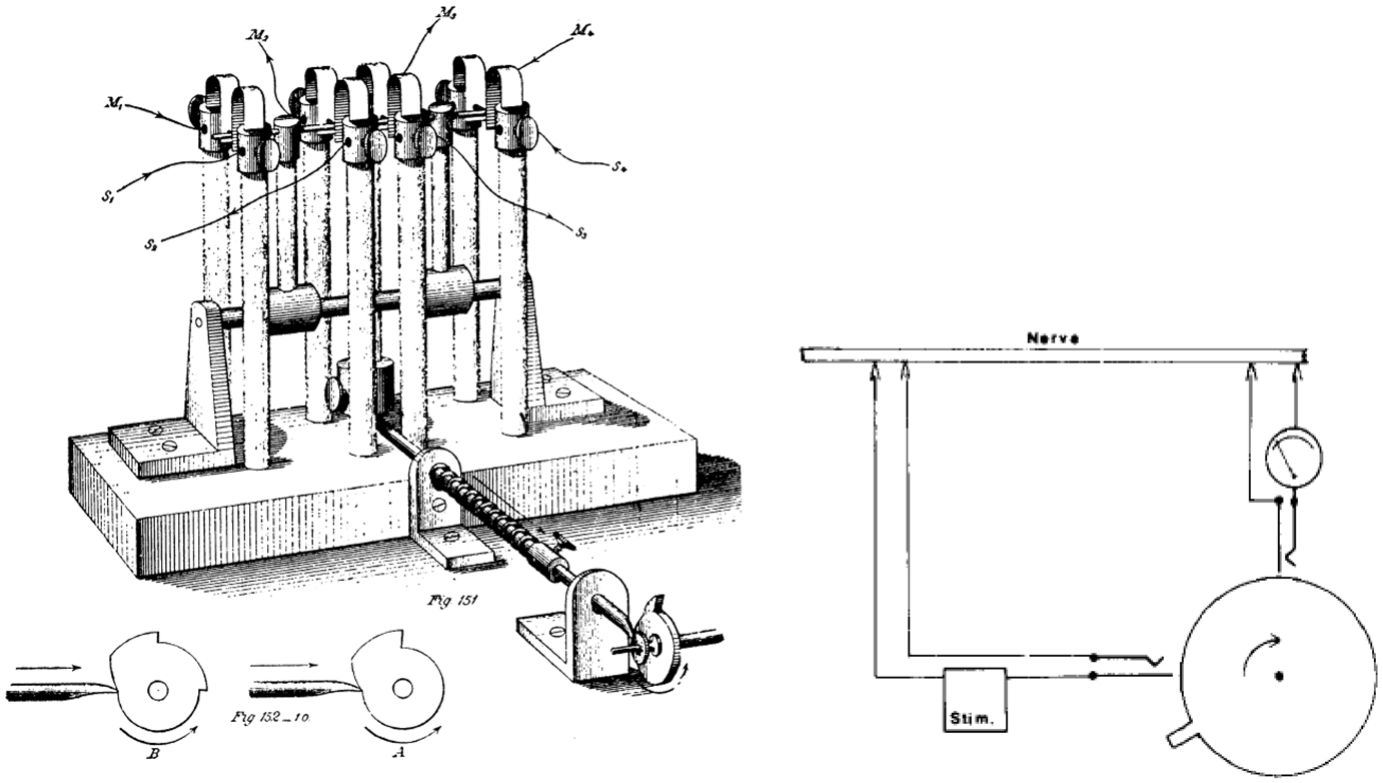
**A** du Bois-Reymond’s (du Bois-Reymond, 1849) design for a rheotome that would deliver a stimulus to a nerve and at a variable time afterwards record the electrical activity with a galvanometer. **B** A schematic of Bernstein’s differential rheotome. From Schuetze (1983)

That du Bois-Reymond qualified the statement in the previous quote, characterizing the negative variation as in “some way intimately related to” rather than asserting that it was the change that caused the contraction in muscle points to a challenge in showing that the two are identical. The best strategy to establish that the two were in fact the same, he decided, was to demonstrate that they moved at the same speed. Helmholtz (1850a, b) had already compared the times it took for nerve impulses to propagate along different lengths of nerve, concluding that they traveled with a speed of approximately 27 m/s. To investigate the time course of the negative variation, du Bois-Reymond developed a rheotome that would allow for stimulating of a nerve during one interval while recording the response at a set distance further along the nerve with a galvanometer (Fig. 14A).

Du Bois-Reymond did not achieve satisfactory results from his rheotome and assigned the task to Bernstein, who developed what he referred to as the *differential rheotome* (Fig. 14B). Bernstein’s invention facilitated administering a stimulus and at different intervals afterwards sampling the nerve response with the galvanometer. When the point of contact for the recording circuit was close to that of the stimulating circuit, no response was detected. Based on multiple measurements on different lengths of nerve segments, Bernstein (1868) calculated that on average the negative variation traveled 28.718 m/s. and concluded that “the process of excitation and the process of negative variation have one and the same speed” (p. 188) and so are identical phenomena.

This process of bringing different experimental practices and their results into “resonance” with one another is another significant event in the life cycle of an epistemic thing, according to Rheinberger (1997). In one sense, Bernstein’s achievement is clear: in demonstrating that the negative variation and nerve impulses travel at the same speed, he provided substantial evidence that two previously distinct phenomena are in fact the same. But Rheinberger suggests that during moments such as this, something even more important has transpired. Researchers have no access to unmediated evidence for the phenomena they seek to characterize and explain; findings come in the form of material traces—inscriptions of one kind or another. Bernstein could not compare the speed of the negative variation to the speed of nervous transmission in some primitive or unmediated fashion, he could only compare it to *another measurement*. “Scientific objects come into existence,” Rheinberger contends, “by comparing, displacing, marginalizing, hybridizing, and grafting different representations with, from, against, and upon each other” (p. 109). It is this “matching” he argues, that bestows “that sense of ‘reality’ we ascribe to the scientific object under investigation” (Rheinberger, 1997, p. 111). This suggests that by measuring the speed of the negative variation (and comparing it to Helmholtz’s results) Bernstein not only identified two previously unique phenomena, but also established the negative variation as a legitimate object of scientific interest—one that could be regarded as a real feature of the natural world.

**Fig. 15:**
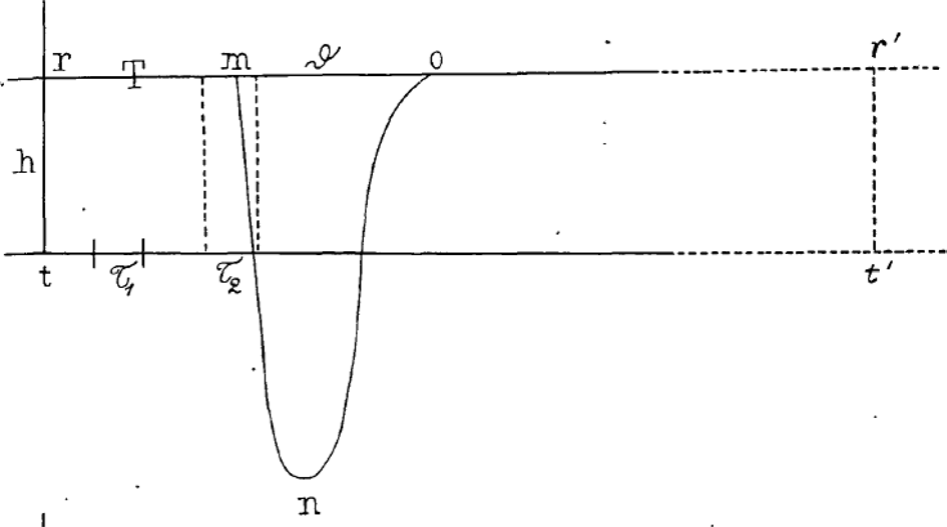
Bernstein’s (1868) graph of the negative variation showing an overshoot of the neutral reading on the galvanometer

Bernstein’s finding succeeded in rendering the negative variation into a phenomenon involved in nerve and muscle transmission. But it also allowed him to develop a more detailed account of the time course over which it became manifest. To do this, he varied the distance between the point of stimulation and point of recording and plotted the resulting current detected by the galvanometer at different times. Figure 15 shows how the current over the interval of one revolution of the rheotome (t–t’) developed after administration of a stimulus beginning at t. *T*_1_, T_2_, represent different sampling periods. T_1_ precedes the negative variation reaching the recording site; the measured current (reflected in the line r–r’) is just du Bois-Reymond’s nervous current (h). At T_2_ the negative variation from the line r–r’ has begun, eventually reaching the peak n before returning to the ongoing nervous current at o.

This figure clearly shows not only that the negative variation constitutes a decrease the nervous current but that the current drops below the line t–t’, which represents a neutral reading on the galvanometer. Bernstein noted this *sign reversal* that subsequently was referred to as the *overshoot*. In 1876 he reported that he only found it in nerve but not when he made similar measurements of the time course of the negative variation in muscle. As discussed by Grundfest (1965), this seemed to concern him. Bernstein states in a footnote: “This seems to contradict the claim that the negative fluctuation in nerves can be stronger than the nerve current. For muscle it is safe to conclude that the negative variation can only be cancelled, and since this cannot be a coincidence, I might ascribe my observation of the nerve to the account of the electrotonus, as a result of the electrical stimulation being too strong, about which, however, further clarification is necessary” (Bernstein, 1876, p. 53). du Bois-Reymond (1875) likewise reported he could not detect the overshoot in muscle, but Burdon-Sanderson and Gotch (1891) eventually did establish the overshoot in muscle.

Bernstein’s research in the 1860s and 1870s served to characterize du Bois-Reymond’s negative variation in far greater detail. First, by demonstrating that the negative variation travelled along nerves at the same rate that Helmholtz had shown nerve impulses to travel, he established that the two phenomena were in fact the same. This was no small feat, given that the methods used by Helmholtz, drawn from ballistics and telegraphy, were quite different from those employed by du Bois-Reymond and Bernstein. To procure the needed data, Bernstein had to develop the differential rheotome. This enabled him not just to determine how fast the negative variation traveled but also to characterize its evolution over time. Looking at Bernstein’s work with modern eyes, it is striking how similar his graph is to standard representations of the action potential used today. Detectable with multiple experimental procedures and richly characterized, with Bernstein’s contribution the negative variation began to assume the status of a phenomenon in the world.

### Resituating the negative variation in the context of the membrane potential

**Fig. 16:**
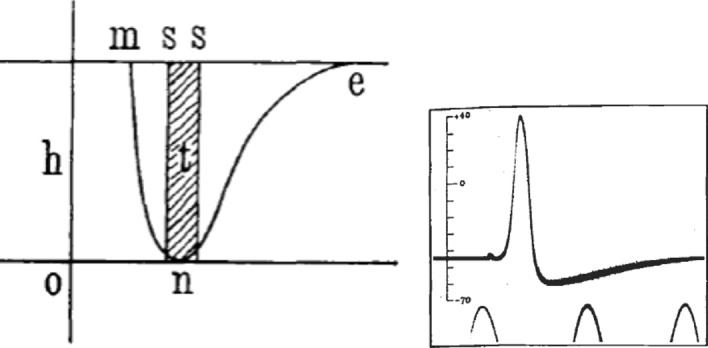
**A** Bernstein’s (1912) representation of the action potential as dropping just to 0. **B** Hodgkin and Huxley’s (1939) tracing of the action potential, showing an overshoot of 0 to approximately + 40mv

Given the initial characterization of the negative variation as a reduction in preexisting currents present in muscle and nerve, Bernstein’s, 1902 recharacterization of these currents as potentials required that he recharacterize the negative variation as well. His subsequent framing proposed that the negative variation occurred when the membrane is disrupted, allowing K^+^ ions to travel from the inside, where they were in higher concentration, to the outside, where they were in lower concentration, constituting a current. When the disruption ceased, the potential difference was reestablished. Accordingly, the negative variation was characterized as the temporary reduction in the membrane potential. Bernstein’s formulation of the membrane potential solely in terms of K^+^ ions, however, had consequences for his earlier characterization of the overshoot. On his new account, the concentration of ions inside the nerve could only drop until it was in equilibrium with that outside, representing a potential of 0: “A consequence of this theory would now be that the negative fluctuation would have to reach a maximum limit, which would be given by the strength of the membrane potential, and that this could not be reversed when stimulated” (1912, p. 105). Accordingly, when Bernstein graphs the negative variation in 1912, he only shows it as declining to 0 (Fig. 16A). He does not mention the overshoot that he had identified in 1868.

For the action potential to overshoot 0, additional ions had to be involved that would determine the current when K^+^ ions reached equilibrium. Indeed, even as Bernstein advanced his proposal, there was evidence that other ions were involved. In 1896 Katz had shown that while K^+^ was in greater concentration inside the cell than outside, Na^+^ was in greater concentration outside than inside. Moreover, in the same volume in which Bernstein advanced his account of the membrane potential, Overton (1902) had found that without Na^+^ in the medium, muscles would not contract when stimulated: “Only the sodium ions are important for the processes of conduction of excitation and muscle contraction, whereas the anions and the undissociated molecules are not involved or at most play a very minor role” (Overton, 1902, p. 368). Overton further asserted that he had had “for the first time demonstrated a specific function of sodium in the vertebrate organism” (1902, p. 380). Given the high concentrations of K^+^ in muscles and Na^+^ in the extracellular fluid, he conjectured that “During muscle contraction or the conduction of excitation a certain exchange must occur between the cations inside the muscle fibers (most likely potassium ions) and the sodium ions in the solution around the muscle fibers” (1902, p. 381).

In the early decades of the twentieth century investigators such as Erlanger and Gasser developed more sensitive electrodes and recording techniques and described the negative variation, which they termed *action currents* in more detail. In the abstract to their talk presented at the American Physiological Society December 1924 Bishop, Erlanger, and Gasser (1925) switched to the term *action potential*.^14^ Despite the name change, researchers continued to adopt Bernstein’s characterization of the phenomenon. The overshoot previously described by Bernstein was forgotten until Hodgkin and Huxley (1939) measured the resting and action potentials in the giant axon of the squid with microelectrodes and found that the action potential exceed the resting potential (Fig. 16B). At that point, as characterized by Grundfest (1965) "the overshoot was, indeed, a new discovery." Hodgkin at that point read Overton’s paper and began to consider the roles of Na^+^ ions in characterizing the action potential. Following upon that, Hodgkin and Huxley (1952) advanced a mathematical model of the action potential in terms of the movement of multiple ions across the membrane. While this was an important advance which provided a mathematical description of action potentials used in many computational models in neuroscience, it was very much in the tradition of Bernstein’s characterization of the negative potential as a reduction of the membrane potential, itself understood as resulting from differential ion concentrations across the membrane.

### Reflections on the discovery of the action potential

Since its first detection by Matteucci, researchers viewed what we refer to as the action potential as a diminishment or reversal of something. For Matteucci it was the termination of the proper current of muscle when the muscle was exhausted in tetanus. For du Bois-Reymond it was the diminution of the muscle or nerve currents. For Bernstein it was the reversal, and later the abolition, of the membrane potential. Starting with du Bois-Reymond, it took on more significance than the mere ceasing of another phenomenon—it was the pulse, the signal, communicated along nerves. Establishing this required developing instruments such as the differential rheotome, which Bernstein employed to establish its identity with the nerve impulse and to graph its time course. So characterized, the negative potential became a central phenomenon for electrophysiologists.

When Bernstein recharacterized the current as a potential due to differential K^+^ ion concentrations inside and outside the nerve, he was forced to deny the overshoot that he had identified in his earlier investigations. Hodgkin and Huxley rediscovered the overshoot, leading them to characterize the action potential in terms of multiple ions. Yet, the account they offered is recognizably a variant on that established by Bernstein.

## Conclusion: what does it take to establish a new phenomenon?

In the case of both the membrane potential and the action potential, a long history of inquiry intervened between when they were first detected and when Bernstein arrived at a conception of them as richly characterized phenomena. While the history of these two phenomena does not provide a universal script for the establishment of phenomena, it does reveal important elements in how researchers move from findings grounded in specific experimental arrangements to characterizing what they take to be regular happenings in the world.

Although sometimes new phenomena are anticipated given existing theoretical frameworks, in many cases they emerge as novelties in the course of investigations. In these instances, the first step is the detection that something is happening that does not have a place in the existing conceptual framework. When Galvani began his inquiry, researchers had already demonstrated that muscles contract in response to electrical stimulation. Even the idea of contracting in response to a release of electricity some distance away was not a novel observation. What was novel was a frog muscle contracting without an external stimulus, electrical or otherwise, but when a nerve and the external surface of the muscle came into contact. Likewise, Matteucci’s detecting a diminution in the proper current in muscle in the tetanic state was novel and not predicted by an existing theoretical framework.

The progression from novel findings to new phenomena in these cases was facilitated by new instruments and the application of them to generate new findings that further characterized the phenomena. We have described the use of instruments such as galvanometers and rheotomes. There was considerable variability in the specific instruments researchers used, but also in the ways they deployed them. For instance, while Nobili, Matteucci, and du Bois-Reymond all used the galvanometer in their investigations, the galvanometers and the protocols for their use varied substantially, including how the frogs were prepared, how many times wire was wound in the galvanometer, how specimens were positioned on the pads of the galvanometer, and even the methods used to induce tetanus.

Corresponding to the differences in experimental procedures, investigators generated diverse findings that over time liberated these phenomena from the specific contexts in which they first appeared. Like the phenomena described by Hacking (1983), Galvani’s findings of an inner source of electricity that could elicit muscle contractions were intimately tied to the particular experimental conditions he employed. Even if he assumed animal electricity was responsible for the muscular contractions of animals outside the laboratory (and it seems he did), the phenomenon, “at least in a pure state,” as Hacking puts it “can only be embodied by [certain kinds of apparatus]” (p. 226). At the outset, the experimental set ups in which each phenomenon could be identified were highly restricted.

Although still always investigated in some experimental context, as researchers developed multiple experimental protocols from which they procured different information, the phenomena began to acquire independence. By using the galvanometer in addition to the galvanoscopic frog, Nobili not only provided an alternative means of accessing the phenomenon but was able to characterize additional features of it: the direction and intensity of the current. Matteucci, and especially du Bois-Reymond, showed that the current flowed from the tendon or, if the nerve were cut, the transverse surface to the outside of the muscle. Using the fall rheotome, Hermann showed that the current grew in strength over the interval after the muscle was injured. By employing an instrument that allowed him to regulate temperature, Bernstein was able to demonstrate that voltage increased linearly with temperature and in this respect fit the Nernst equation. In these ways, animal electricity was “recursively constituted” as the membrane potential, undergoing a process of gradual redefinition through its recurrence in a succession of evolving experimental contexts (Rheinberger, 1997, p. 76). The development of new instruments not only afforded researchers with novel ways to intervene experimentally, but also provided new spaces of representation. It is through these different kinds of inscriptions (e.g., Bernstein’s graph of the time course of the negative variation, du Bois-Reymond’s descriptions of the deflection of the galvanometer) that epistemic things come to be embodied; they are the “material forms of the epistemic things under investigation (Rheinberger, 1997, p. 106). Crucially, graphical representation facilitates the kind of disembedding discussed above, allowing the targets of inquiry to be “re-presented outside their original and local context and inserted into other contexts” (Rheinberger, 1997, p. 106). As a result of this process (and concomitant experimental investigations), animal electricity gradually came be to the richly described membrane potential.

The same process of accumulating additional evidence occurred in the case of the action potential. Matteucci did not present much evidence, and in fact seemed unsure of whether the reduction in current was a significant phenomenon at all, but du Bois-Reymond combined the use of the galvanometer and the rheoscopic frog to make his case that each stimulation resulted in a negative variation. He also created a means to detect the negative variation by alternating the direction in which a nerve was stimulated and showing successive reversals in two galvanometers responding to parts of the nerve on opposite sides of the point of stimulation. Bernstein took the further step of measuring the speed with which the negative variation travels and showing the time course in which the change in current developed. In doing so, he compared results obtained using the differential rheotome with Helmholtz’s measurement of the speed of nervous transmission, bringing distinct experimental practices and representations into resonance with one another. Consilience between representations generated in distinct contexts (i.e., different “spaces of representation”) is what, Rheinberger suggests, undergirds our sense that a given phenomenon is real (p. 111).

Jointly, the concepts of *recursive constitution* and *resonance*, in concert with the details of the history recounted above, provide the resources required to address the tension Feest identified between the differing conceptions of phenomena espoused by Hacking and Bogen and Woodward. Experimental systems are, as Rheinberger (1997) notes, “necessarily localized and situated generators of knowledge” (p. 76). Likewise, phenomena begin their lives embedded in such experimental setups (i.e., the technical objects that define a given experimental arrangement), isolated and constrained by the conditions that support their investigation. However, neither the experimental system nor the epistemic objects they contain are static entities. As an experimental system is reproduced, this allows for, or indeed necessitates, that the scientific objects under investigation are recursively constituted. If recursive constitution opens the door for the kind of disembedding that we have argued is crucial to bridging the gap between Hacking and Bogen and Woodward, resonance provides the other crucial ingredient. Resonance involves a bringing into harmony or negotiation of disparate experimental practices and representations. The outcome of these kinds of comparisons and displacements, when they are successful, is the sense we have that a given phenomenon is “real,” or as Bogen and Woodward put it, part of “the natural order itself.”

The heterogeneity of findings thus both contributes to the extraction of the phenomena from the specific arrangements in which they were first detected but also present the new challenge of synthesizing a coherent characterization of the phenomena from them. For Galvani, it sufficed to analogize muscle to the Leyden jar to characterize its electrical activities. While investigators from Nobili to du Bois-Reymond focused on what they assumed was a preexisting current in muscle and nerve, Hermann challenged this claim, developing procedures that indicated that current only arose after injury to the tissue. Bernstein, however, maintained a focus on was preexisted in the tissue before injury, and introduced the understanding of a membrane potential resulting from differential ion concentration and characterized by the Nernst equation. This became the standard characterization of the membrane potential.

In the case of the negative variation, du Bois-Reymond had already begun to develop a characterization of the phenomena that dislodged it from the condition of tetanus in which he, following Matteucci, was able to produce it in the laboratory. He viewed tetanus merely as revealing a reduction in the muscle or nerve current in response to each stimulation. In his early work measuring the velocity with which the negative variation traveled along the nerve and demonstrating its time-course, Bernstein simply adopted du Bois-Reymond’s conceptualization of it as a reduction in the ongoing current. Once he replaced the current with a potential, he could then view the negative variation as itself a current resulting from the opening of the membrane to the flow of ions.

An important part of setting out a new phenomenon is to provide a distinctive name or label for it. The name a researcher chooses often suggests a particular understanding of the phenomenon. Galvani referred to the current he identified as *animal electricity*. This very general term reflects a limited understanding of what he detected, but it did serve to link it to other electrical phenomena such as the activities of electric fish. Nobili referred to the *frog current* or the *proper current*; the latter term that was adopted by Matteucci. Demonstrating a current in both muscle and nerve, du Bois-Reymond introduced terms specific to each: the *muscle current* and the *nerve* current. As he contended that these currents were only found once the muscle or nerve had been injured, Hermann adopted the terms *injury current* and *demarcation current* to signal that they did not exist except when muscle or nerve was injured. Once he argued that what preexisted was an ion gradient that produced an electrical potential, Bernstein adopted the term *membrane potential*, which has become the standard term.

A similar history occurred with the action potential. When Matteucci first encountered it as a reduction of the proper current in tetanus, he did not offer a special name. That perhaps signals that it was a finding he found worth noting, but not of primary importance. Du Bois-Reymond, on the other hand, named it the *negative variation*, descriptive of the fact that it appeared as a reversal of the muscle or nerve current. Moreover, tetanus was just a means of demonstrating something he maintained happened more generally as nerves and muscles transmitted activity. This was the name maintained by Bernstein even as he offered a new account of what it was negative with respect to—the membrane potential. Bernstein’s continuation of the name adopted by du Bois-Reymond is perhaps a reflection of his overall loyalty to his mentor. But recognizing that the phenomena had been extracted from the original context in which du Bois-Reymond had named it, subsequent researchers renamed it the *action current* and eventually the *action potential*.

This untidy history of different researchers each adopting their own favored vocabulary is reminiscent of the kind of geopolitical game of musical chairs epitomized by the history of border towns in regions like Alsace-Lorraine. While the roads, buildings, and citizens may remain largely unchanged, the political organization, the currency people use, perhaps the languages they speak, and how they relate to those beyond their borders, can change over time. In conjunction with these changes, the names of towns, cities, and occasionally entire regions may be changed depending on what flag is flown. Instead of physical land, in science it is the findings generated in the laboratory that remain largely unchanged and the conceptual frameworks scientists use to characterize them that change. Even when recognizing that their phenomenon corresponds to what others had reported previously, researchers adopt new names as they discover new features of the phenomenon or characterize it differently.

We have argued that over the course of successive investigators, both the membrane potential and the negative variation came to be accepted as features of muscles and nerves, not just interesting experimental findings. One measure of this shift is how effective arguments against previous findings are in leading researchers to reject a phenomenon as an artifact. The contrasting responses to Volta’s challenge to Galvani and Hermann’s challenge to du Bois-Reymond are illustrative. By arguing that the two metals Galvani’s used in his initial experiments were themselves responsible for the electrical current, Volta argued against not just Galvani’s interpretation of his data, but the existence of the phenomenon (an endogenous current) altogether. Even though Galvani was able to replicate the phenomenon without using any metals, animal electricity was widely regarded as an experimental artifact for over two decades. When Hermann showed that muscle currents only arose after injury, he did not reject the phenomenon as an experimental artifact. Rather, he recharacterized it as an injury current. In other words, he still regarded the current as an object of scientific interest and retained much of du Bois-Reymond’s account of it. He simply limited it to the context in which muscle was injured. In Galvani’s case, the phenomenon was only manifest in a specific experimental design, and to cast doubt on the integrity of the design was therefore to undermine the only real source of evidence for it. In contrast, by the time Hermann challenged du Bois-Reymond’s claim about the current, a wide range of investigations into the phenomenon had been conducted by numerous individuals using a variety of means and a number of features were attributed to it. Even as he denied that the current preexisted injury, the case for something significant happening was strong; accordingly, Hermann did not reject the phenomenon outright.

Moreover, Hermann left open the question of what preexisted the injury. We have not discussed Hermann’s attempt to answer this question in terms of the chemistry of nerves and muscles. We focused instead on how Bernstein, by invoking work on ion concentrations across membranes, advanced an alternative electrical characterization of what preexisted—a potential, not a current. In terms of that, the negative variation was recognized as the current transmitted along nerve and muscle. Even though subsequent researchers modified the details as to which ions contributed to the membrane potential and the negative variation/action current, they did not change the basic understanding of the phenomena. With Bernstein both phenomena had been established as features of nerves and cells.

There is a final feature of this history of research worth noting: research initially focused on just one phenomenon—a current in muscle and nerve. In the end, two interrelated phenomena were distinguished. Looking backwards, one can see that aspects of both the membrane potential and action potential were present in early accounts of animal electricity. But both were described in terms of current, the negative variation being a variation in a current, obscuring the distinction. When Hermann’s finding sparked Bernstein to better characterize what is constant in muscles and nerves, he identified the membrane potential, which is treated as enduring until the nerve is stimulated. The negative variation, by contrast, is distinctively dynamic. Insofar as the negative variation is a change in the otherwise constant membrane potential, the interrelations of the phenomena became clear: the negative variation, as Bernstein conceived of it, depends on his characterization of the membrane potential. The characterization of the latter provides the framing for the former.

In recent years a number of philosophers have directed their attention to characterizing phenomena. The diachronic perspective adopted here captures features of several of their accounts. As we have emphasized throughout, the phenomena on which we have focused began their lives embedded in particular experimental activities, as emphasized by Hacking, but as additional techniques gave rise to new findings and conceptualizations of what was happening, these phenomena acquired the independence and regularity Bogen and Woodward describe. Likewise, Feest emphasizes not just the experimental procedures but also the significance of the conceptual framing of phenomena in characterizing what she distinguishes as surface and hidden phenomena. However, by adopting a diachronic perspective when considering the historical trajectories of these research programs, we obviate the need to invoke two types of phenomena. Rather, we can appreciate how phenomena are gradually extricated from specific experimental protocols as an increasing variety of techniques and procedures target what researchers regard as the same phenomena, enabling richer and more robust characterizations to emerge. While phenomena are still generated in particular experimental protocols, the multiplicity of techniques and descriptors brought to bear militate towards recognizing them as regular happenings in the world. Finally, although the contributors to the research often advanced mechanistic proposals to explain the phenomena, the manner in which the phenomena were successively reconceptualized stands apart from how they are explained (a featured emphasized by Colaço, 2018). What is crucial is developing multiple experimental approaches, employing them to generate diverse findings, and then putting these together in characterizations of what is happening in the world.

## Footnotes

1 More attention has been directed at how conceptions of phenomena are revised (see Bechtel & Richardson, 1993/2010; Colaço, 2018, 2020; Kronfeldner, 2015).

2 Although, as far as we are aware, philosophers of science have not investigated these early developments in electrophysiology, both historians and scientists have analyzed important episodes in this history. Of particular note is Lenoir’s (1987) synthetic account of the history that inspired our inquiry. One difference of focus in our analyses is that Lenoir integrated the development of the experimental instruments with the mechanistic explanations each investigator advanced. We do not discuss the often-speculative mechanistic accounts of the various investigators since we think those can be separated from their contributions to the establishment of the phenomena.

3 Frogs exhibit some, but not all, the features that Ankeny and Leonelli (2011, 2020) ascribe to model organisms. Frogs were viewed as instantiating the same nerve and muscle tissues as other animals, including vertebrates, and so served as representations of this broader class. As we will see, Galvani developed a way of preparing frogs for research that was widely adopted (it forms part of what Ankeny and Leonelli characterize as a repertoire). But, other features emphasized by Ankeny and Leonelli do not apply: there was no standardization of the frogs themselves (they were captured from the wild, not ordered from a common source) and there was no knowledge of their genetics, let alone tools for manipulating them genetically.

4 Piccolino and Bresadola (2013) provide a detailed history of Galvani’s research, showing that the experiments reported in *De viribus electricitatis* are selected from a vast number of experiments Galvani conducted starting in 1780.

5 Throughout the periods discussed in this paper, current was understood to flow from the positive pole to the negative.

6 For a historical analysis of Matteucci’s investigations, see Moruzzi (1996).

7 For a detailed biography of du Bois-Reymond, see Finkelstein (2013).

8 The discussion below is based on du Bois-Reymond (1852), a translated, edited, and abbreviated version of the first two volumes of *Untersuchungen über thierische Elektricität* (du Bois-Reymond, 1848–1884).

9 The translations of non-English texts, unless attributed to a translation, are our own.

10 The gas constant is a constant for all gases that relates energy (volume and pressure of the gas) to the temperature and the number of moles of the gas. The Faraday constant is the electrical charge of one mole of electrons. The valence of an ion is the number of electrons gained or lost to complete a full orbit and that can be transferred to another atom in creating an ionic bond between them.

11 As De Palma and Pareti (2011) demonstrate, a number of other theorists were applying the account of ionic gradients to nerve and muscle activity, including Chagovec, Oker-Blom, and Boruttau. There is no evidence that Bernstein drew upon these sources in developing his membrane theory.

12 This is also how du Bois-Reymond interpreted Matteucci’s writings about his finding: “He also admitted, regarding the supposed effect of the tetanic state in diminishing the current, that he did not intend his observations to apply to the state of the current during the contraction itself: but to the injurious effect of long continued contractions on the life of the muscle, and through this, upon its electric action.” (p. 128).

13 The use of the rheoscopic limb in conjunction with the galvanometer in this context supports the claim made by Rheinberger (1997) that “the generation of new phenomena is always and necessarily coupled to the coproduction of already existing ones” (p. 75). Continuities such as this support the reproduction of an experimental system and provides a necessary link between otherwise isolated experimental setups.

14 In a second paper at the same conference the authors retained the term *action current*. They also offer no clarification of why they switched to *action potential.* The following year both talks by this group referenced only action potentials. Davis (1926) states “This is termed the ‘action potential’ by Erlanger and Gasser (1924) when recorded by an instrument which draws no current, corresponding to the familiar ‘action current’ for the current which flows through a galvanometer.” However, Erlanger and Gasser (1924) do not actually use the term *action potential*, only *action current*.
